# Oxygenation and intestinal perfusion and its association with perturbations of the early life gut microbiota composition of children with congenital heart disease

**DOI:** 10.3389/fmicb.2024.1468842

**Published:** 2025-01-15

**Authors:** Hanna Renk, Ulrich Schoppmeier, Jennifer Müller, Vanessa Kuger, Felix Neunhoeffer, Christian Gille, Silke Peter

**Affiliations:** ^1^Department of Neuropediatrics, Developmental Neurology and Social Pediatrics, University Children’s Hospital Tübingen, Tübingen, Germany; ^2^German Centre for Infection Research (DZIF), Partner Site Tübingen, Tübingen, Germany; ^3^Department of Pediatric Cardiology, Pulmonology and Pediatric Intensive Care Medicine, University Children’s Hospital Tübingen, Tübingen, Germany; ^4^Institute of Medical Microbiology and Hygiene, University of Tübingen, Tübingen, Germany; ^5^NGS Competence Center Tübingen (NCCT), University of Tübingen, Tübingen, Germany; ^6^Department of Neonatology, Heidelberg University Children’s Hospital, Heidelberg, Germany

**Keywords:** congenital heart disease, intestinal microbiota, next-generation sequencing, oxygen to see, O2C, oxygenation, gut microbiome

## Abstract

**Background:**

Early life gut microbiota is known to shape the immune system and has a crucial role in immune homeostasis. Only little is known about composition and dynamics of the intestinal microbiota in infants with congenital heart disease (CHD) and potential influencing factors.

**Methods:**

We evaluated the intestinal microbial composition of neonates with CHD (*n* = 13) compared to healthy controls (HC, *n* = 30). Fecal samples were analyzed by shotgun metagenomics. Different approaches of statistical modeling were applied to assess the impact of influencing factors on variation in species composition. Unsupervised hierarchical clustering of the microbial composition of neonates with CHD was used to detect associations of distinct clusters with intestinal tissue oxygenation and perfusion parameters, obtained by the “oxygen to see” (O2C) method.

**Results:**

Overall, neonates with CHD showed an intestinal core microbiota dominated by the genera *Enterococcus* (27%) and *Staphylococcus* (20%). Furthermore, a lower abundance of the genera *Bacteroides* (8% vs. 14%), *Parabacteroides* (1% vs. 3%), *Bifidobacterium* (4% vs. 12%), and *Escherichia* (8% vs. 23%) was observed in CHD compared to HCs. CHD patients that were born by vaginal delivery showed a lower fraction of the genera *Bacteroides* (15% vs. 21%) and *Bifidobacterium* (7% vs. 22%) compared to HCs and in those born by cesarean section, these genera were not found at all. In infants with CHD, we found a significant impact of oxygen saturation (SpO2) on relative abundances of the intestinal core microbiota by multivariate analysis of variance (*F*[8,2] = 24.9, *p* = 0.04). Statistical modeling suggested a large proportional shift from a microbiota dominated by the genus *Streptococcus* (50%) in conditions with low SpO2 towards the genus *Enterococcus* (61%) in conditions with high SpO2. We identified three distinct compositional microbial clusters, corresponding neonates differed significantly in intestinal blood flow and global gut perfusion.

**Conclusion:**

Early life differences in gut microbiota of CHD neonates versus HCs are possibly linked to oxygen levels. Delivery method may affect microbiota stability. However, further studies are needed to assess the effect of potential interventions including probiotics or fecal transplants on early life microbiota perturbations in neonates with CHD.

## 1 Introduction

Congenital heart defects (CHD) are the most common birth defects affecting infants worldwide (Dastgiri et al., 2002; Dolk et al., 2010; van der Linde et al., 2011). In early life, neonates affected by a CHD are frequently exposed to a “hostile environment” when requiring admission to Pediatric Intensive Care Units (PICU). Adverse physiology in these critically ill neonates, antibiotic treatment and hospital-acquired pathogens may impact the balance, development and function of the gut microbiota (Wijeyesekera et al., 2019). Colonization of mucosal tissues during infancy is crucial in maintaining host metabolic and immune homeostasis and early life microbiota is known to shape the immune system (Gensollen et al., 2016; Reynolds and Bettini, 2023). Thus, disruption of intestinal host-commensal interactions in this “window of opportunity” may critically influence health and disease outcome of infants with CHD (Renz et al., 2018).

In general, the establishment of an infant’s early life intestinal microbiota is a complex process that includes a variety of influencing factors. Maternal-child microbial seeding as well as ecological forces and environmental conditions contribute to the establishment of an infant’s intestinal microbiota (Enav et al., 2022). The first and most important influence on a newborn’s microbiota is vertical transmission of maternal flora. Vaginal delivery creates an intestinal microbiota that strongly correlates with the mother’s vaginal flora which contains *Lactobacillus* and *Prevotella* species. In contrast, infants born by cesarean section bear their mother’s skin commensals, show a lower diversity of microbiota, lower *Bifidobacterium* and *Bacteroides* species and a higher number of *Clostridium difficile* (Penders et al., 2006; Matamoros et al., 2013). Additionally, infant feeding has been demonstrated to play a major role in early colonization of the infant gut. Formula feeding causes a different intestinal flora compared to breast milk, characterized by increased diversity of species and overrepresentation of *C. difficile* (Azad et al., 2013), *Escherichia coli* and *Bacteroides* species (Penders et al., 2006).

Whether and how the intestinal microbiota of term neonates with CHD differs from that of healthy neonates and may predispose to particular pathogenic conditions including necrotizing enterocolitis (NEC) or nosocomial infections, is unclear. Only few studies characterized the microbiota that colonizes neonates with CHD shortly after birth (Ellis et al., 2010; Huang et al., 2022; Liu et al., 2022; Magner et al., 2023; Toritsuka et al., 2024; Zhang et al., 2023). Zhang et al. revealed an increase in pathogenic bacteria and a decrease in diversity and richness of the intestinal microbiota in infants with heart failure and CHD compared to healthy controls (Zhang et al., 2023). Furthermore, deep metagenomic sequencing and metabolomic profiling of the intestinal microbiota of 45 neonates with critical CHD suggested an aberrant gut microbiome that is associated with metabolomic perturbations compared to healthy controls (Huang et al., 2022). This could lead to immune imbalance and adverse clinical outcomes in neonates with critical CHD.

Intermittent hypoxia, e.g., in obstructive sleep apnea syndrome, can reduce oxygen supply of the intestinal mucosa, alter gut microbiota diversity and affect the intestinal epithelial barrier (da Silva et al., 2024; Moreno-Indias et al., 2015). Moreover, intestinal microbiota alteration has been linked to the epithelial damage following intestinal ischemia–reperfusion injury (Wang et al., 2012). This underpins that hypoxia to the gut and reduced systemic perfusion in neonates with CHD may both be stressors that affect the normal gut flora and contribute to dysbiosis and intestinal epithelial barrier dysfunction (EBD). EBD facilitates bacterial translocation and increases the risk for post-operative sepsis and organ dysfunction (Feng et al., 2021).

Neonates and infants undergoing cardiopulmonary bypass surgery (CPBS) for repair or palliation of CHD are at increased risk for intestinal injury, inflammation and EBD compared to subjects undergoing control surgeries (Salomon et al., 2021). An abnormal intestinal mileu, including reduced cardioprotective metabolites like short-chain fatty acids have also been linked to feeding intolerance after CPBS (Owens et al., 2024). Interestingly, there is not only evidence for exacerbation of dysbiosis and EBD after CPBS in pediatric CHD, but also for loss of intestinal epithelial integrity already pre-operatively (Salomon et al., 2021; Typpo et al., 2015).

Administration of probiotics to correct dysbiosis and alleviate symptoms of intestinal barrier dysfunction has already been proposed (Ellis et al., 2010). Only recently, probiotics were able to correct dysbiosis induced by cardiopulmonary bypass in infants with CHD, but could not substantially decrease the intestinal damage induced (Toritsuka et al., 2024). Taken together, these studies suggest that neonates with CHD are affected by perturbations of the intestinal microbiota in early life. However, underlying mechanisms are unclear and findings are based only on a limited number of patients. Furthermore, the interplay between potentially influencing factors like hypoxia and altered intestinal perfusion and the gut microbiota of neonates with CHD remains poorly understood (Han et al., 2021; Munley et al., 2023; Xing et al., 2018).

In the present study, we characterized the early life intestinal microbiota composition of neonates with CHD compared to healthy controls. We hypothesized that neonates with CHD would have baseline microbiome alterations, that could be associated with other factors like mode of delivery, oxygenation and intestinal perfusion. Different approaches of statistical modeling were applied to analyze the impact of influencing factor on variation in species composition.

## 2 Methods

### 2.1 Study population

This study was conducted at the PICU and the Maternity ward of the University Hospital, Tübingen, Germany. Ethics approval was obtained from the Medical Faculties’ independent ethics committee (Project number 496/2018BO1). Full-term neonates admitted to the PICU, the patient group (CHD) and healthy controls (HC) on the maternity ward were enrolled between September 2019 and December 2020. Eligibility criteria were either healthy, term born neonates on the maternity ward (HC) or neonates that were admitted to our PICU because of CHD and were planned to undergo cardiopulmonary bypass surgery within the first 4 weeks of life. Neonates with gastrointestinal comorbidity (e.g., congenital abdominal defects, malrotation, need for any gastrointestinal surgery or tube placement etc.) or primary immunodeficiency were excluded. Informed consent to participate was obtained prior to participation from all parents or legal guardians on behalf of their infants.

### 2.2 Fecal sample collection

Fecal samples were collected from neonates with congenital heart disease on the PICU and healthy neonates on the maternity ward during their first 14 days of life. All samples were collected while neonates were physically in hospital. A spoonful of fecal material was collected from the diaper either by the patient’s nurse in charge (on the PICU) or by the mother (on the maternity ward). In healthy neonates from the maternity ward, a single biological replicate was obtained. In neonates with congenital heart diseases, at least 2(−3) biological replicates either from the same stool sample or from a consecutive stool sample were obtained. Samples were collected in DNA/RNA Shield Fecal Collection tubes (Zymo Research) and shaked to ensure proper stabilization. These collection tubes are designed for the collection and preservation of nucleic acids from stool specimens, ensure that fecal material is stable at ambient temperature, and can be frozen for longer-term storage. Samples were placed into the refrigerator at ambient temperature (4°) for up to 6 weeks. Once received in the laboratory, samples were homogenized and 2–5 mL Aliquots were stored at −80°C prior to extraction.

### 2.3 DNA extraction, library preparation, and shot-gun metagenomic sequencing

Genomic DNA was extracted using the ZymoBIOMICS DNA/RNA Miniprep Kit (Zymo Research). For each sample extraction process we used a minimum of 4 and a maximum of 8 aliquots (technical replicates). In brief, samples were bead-beated using ZR BashingBead^™^ Lysis Tubes. Lysis buffer was added 1:1 to 1:2 and the sample was purified via spin-column and filtered to remove PCR inhibitors. Purified DNA was quantified with a Qubit fluorometer using the Invitrogen Qubit DNA Broad Range (BR) Assay Kits. In case of low DNA content, left-over aliquots from the same sample were extracted additionally to increase DNA yield. The samples were stored at −80°C for preservation before further PCR amplification and sequencing.

### 2.4 DNA sequencing

Genomic DNA was extracted and sheared by Covaris M220 (Covaris, Woburn, USA), followed by the construction of a sequencing library TruSeq Nano DNA HT Kit (Illumina, San Diego, USA). Shot-gun metagenomics sequencing was done on an Illumina NextSeq (Illumina, San Diego, USA) with paired end reads (150 bp). The approximate output per sample was 5 GB.

### 2.5 Quality control

To improve the quality and reproducibility of our extraction process, we used a ZymoBIOMICS Microbial Community Standard sample (MOC) alongside each single extraction process. The microbial standard is a well-defined, accurately characterized mock community consisting of Gram-negative and Gram-positive bacteria and yeast with varying sizes and cell wall composition. The wide range of organisms with different properties enables characterization, optimization, and validation of lysis methods such as bead beating. We used this standard to assess and improve the performance of the entire metagenomic workflow. Box whisker plots, including median and interquartile range of relative abundance of the MOC genera of the 12 MOC samples can be found as Supplementary Figure 1.

### 2.6 Bioinformatic analysis

For a first impression of the data we performed a quality control with our in-house nextflow pipeline, which summarizes the output of *fastp (v0.20.1)*. Afterwards, rough taxonomic composition was analyzed with *kraken2 (v2.0.9beta)*. For *kraken2* we used the *k2_pluspf_16gb_20210127* database with default parameters. In the main analysis we first trimmed, and filtered the data with *kneaddata (v0.10.0)*, that uses *trimmomatic (v0.39)* for quality trimming and *bowtie2 (v2.4.5)* for host and spike-in removal. We used the serial mode of kneaddata to remove all reads mapping to human, *Allobacillus halotolerans*, or *Imtechella halotolerans* (used as spike-in organisms). The remaining reads were used for a mapping with *diamond (v2.0.13)*. For diamond we used the blastx option with the fast mapping mode and a block-size (−b) of 15. As reference database we used the actual NCBI nr database (downloaded 08.2022). The diamond result was postprocessed with the daa-meganizer and the Megan mapping file from January 2021. These files were analyzed with *Megan (v6.20.19)*. Sources of the tools, including versions and download Links are available as Supplementary Table 1.

### 2.7 Blood flow and tissue oxygenation

Neonates with CHD underwent pulse oximetry, a noninvasive and continuous monitor of arterial oxygenation, to assess SpO2. This is a commonly used optical technique based on differences in light absorption spectra of oxygenated (OHb) and deoxygenated (RHb) hemoglobin (Nitzan et al., 2014). “Oxygen to see” (O2C) and intestinal blood flow measurements were performed in stable hemodynamic and respiratory conditions within 24 h after the stool sample was obtained. Hemodynamic and respiratory conditions were defined as stable if the heart rate and arterial blood pressure did not change significantly for a period of at least 30 min under the current inotropic and vasoactive medication, no fluid therapy was necessary, there was no bleeding requiring transfusion and normal arterial CO2 levels and sufficient oxygenation was provided. O2C is a multiple channel system that allows non-invasive real-time determination of tissue perfusion and oxygenation. The technology is based on the combination of tissue spectrometry and laser Doppler flowmetry and has been described in detail in previous studies (Klein et al., 2011; Li et al., 2017; Neunhoeffer et al., 2016). The flat probe (LF-3-023 O2C, O2C; LEA Medizintechnik, Giessen, Germany) for combined measurements of relative regional liver Flow (rlFlow) and regional liver oxygen saturation (rlSO2, %) transmits white light (500–800 nm, 1 nm resolution, <30 mW) and continuous wave laser light (830 nm, <30 mW) to the tissue, where it is scattered by the probe and recollected. The spectral components of the collected light are analyzed in comparison with prerecorded deoxygenated and oxygenated hemoglobin, allowing the calculation of tissue oxygen saturation (Klein et al., 2011; Li et al., 2017; Neunhoeffer et al., 2016).

Calculation of relative blood flow (arbitrary units [AU]) is based on Doppler shift of the continuous Doppler wave laser light, which correlates with blood flow velocity and number of moving erythrocytes in tissue (Klein et al., 2011). After abdominal ultrasound, O2C measurements of the liver were performed. The probe was placed in the midclavicular line above the liver, provided by sonography. After a stabilization period of 20 s, measurements were performed as soon as values were stable and results were averaged to obtain regional oxygen saturation (SO2) and regional flow for final analysis. The same method was used in the periumbilical region to obtain intestinal O2C parameters. Intestinal fractional tissue oxygen extraction (iFTOE) was calculated using the equation: iFTOE = [SpO2 (%) – rlSO2 (%)]/SpO2 (%) (Chen et al., 2023; Chock et al., 2023; Schat et al., 2019). Global gut perfusion [ml/min] was calculated using the equation: Average Blood flow velocity [cm/s] * ((Diameter of the portal vein [cm]/2)^2^ * π) * 60 [sec]. To allow for interindividual comparison of this parameter, Global gut perfusion was normalized according to the body weight of the child.

### 2.8 Statistical analysis

Demographic and clinical data were collected via an eCRF in Epi Info^™^ and exported into Microsoft Excel^™^ 2016. Statistical analyses were performed using R version 4.3.1 (R Core Team, 2023). Results of descriptive statistics are either presented as numbers and percentages, mean (SD) for continuous variables or median and range for categorical variables. Due to the mainly descriptive nature of the data, *p* values were not calculated for intergroup comparison of the data. Bar plots were created in order to characterize the intestinal bacterial composition of neonates with CHD and HC. Appropriate data was selected and information about way of delivery was matched. To display information about the core microbiota we normalized absolute abundances to get relative frequencies and calculated means of relative frequencies for each genus. Last, we selected the largest ten genera and renormalized after selection. A detailed description of the method, including R packages used and R scripts can be found in Supplementary Methods 1.

Compositional data analysis was performed by merging data on gene abundance of neonates with CHD and HC with information about way of delivery. Gene abundances were normalized in the common way, so that the abundances for every case summed up to one (relative abundances). Data grouped either by controls and patients or by controls, patients and way of delivery (Gerald van den Boogaart and Tolosana-Delgado, 2013; Egozcue and Pawlowsky-Glahn, 2005; Gloor et al., 2017). The following alpha diversity measures were calculated: Shannon index, Simpson index and inverted Simpson index (Supplementary Methods 2).

Next, we established a formula in order to model the relative bacterial composition as a function of the amount of SpO2 and of the method of delivery. Details about the formula can be found in Supplementary Methods 3. We selected the patients’ data at the first visit. After a first provisional normalization we calculated the mean value for each genus and selected the nine largest ones. This was followed by a second normalization now taking only the nine genera into account. Data were merged with information about oxygenation and about way of delivery. Compositional data were isometric log-ration-transformed (ilr-transformed) and linear regression was performed. The assessment of the model was performed using ANOVA. Composition was then predicted for a range of oxygenation resulting in ilr-transformed data. This was retransformed to relative abundances (for detailed methods of linear models, see Supplementary Methods 3).

Last, we analyzed the relevance of relative abundances of genera to identify naturally occurring clusters of intestinal microbiota composition of neonates with CHD. The following steps were performed: Data of absolute abundances were read from tables followed by imputation and normalization of the data to obtain relative abundances. For the cluster analysis an isometric log-ration transformation (ilr-transformation) was performed followed by a cluster analysis. Heat maps of relative abundances were created and plotted genera were limited to those that resulted in a relative abundance of at least 0.1. Heatmap and the dendrogram resulting from the cluster analysis were combined. Finally, box whisker plots were created for several clinical parameters (e.g., SpO_2_). These plots were grouped by the three clusters identified after the assignment of cases to final clusters (for detailed methods of cluster analysis, see Supplementary Methods 4). Sequencing data of this study are available in the European Nucleotide Archive (ENA)^1^ under the project accession number PRJEB76913. All other data and R Scripts are available in GitHub under.^2^

## 3 Results

### 3.1 Baseline characteristics of study participants

**Figure 1:**
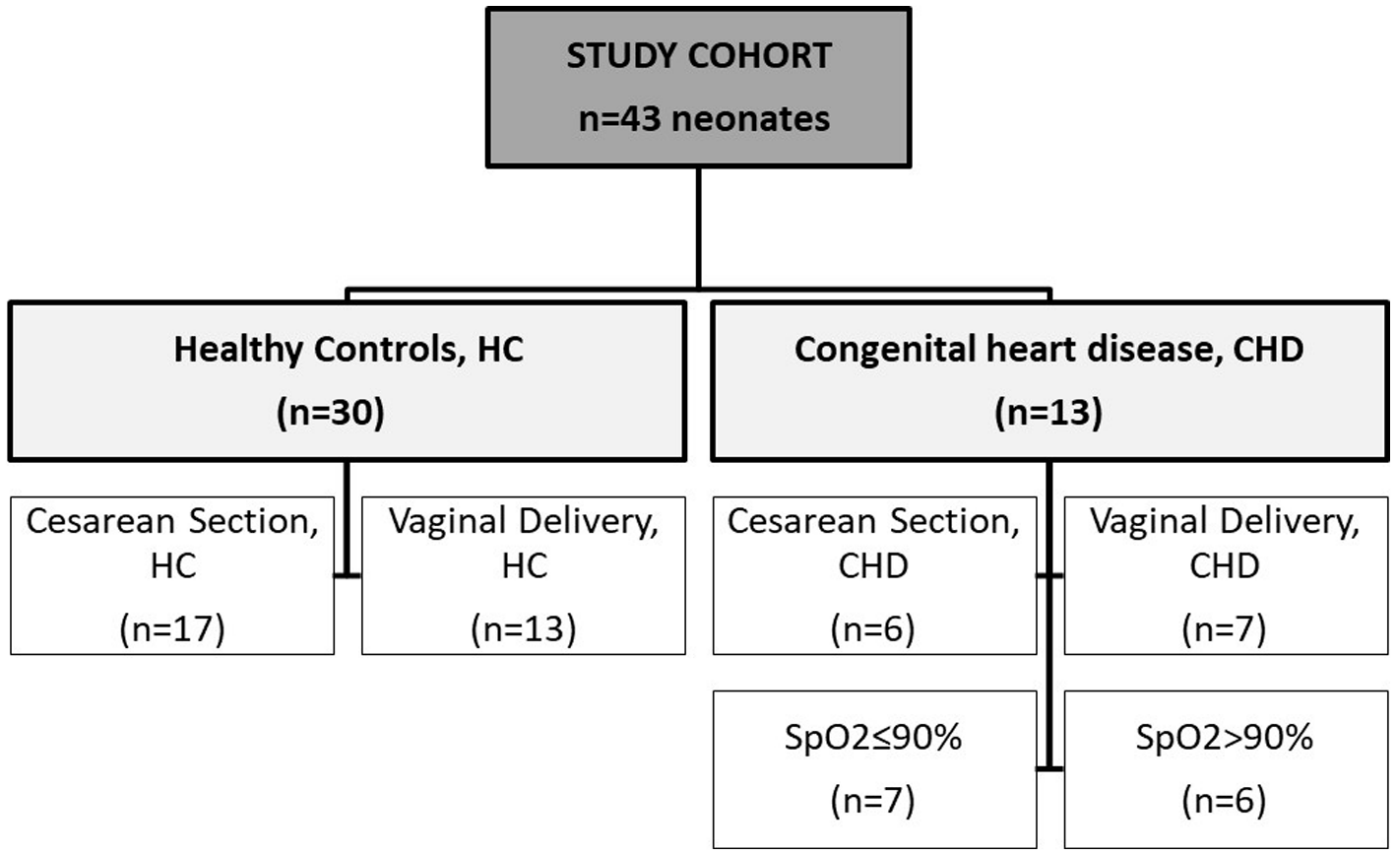
Description of cohort. Samples from 43 neonates were analyzed within this study. Groups were divided into healthy controls (HC) and neonates with congenital heart disease (CHD). Two other independent subgroups were created separately according to mode of delivery (HC and CHD) and according to oxygen saturation (SpO2) of ≤90% or > 90% (CHD group only).

A total of 13 full-term neonates diagnosed with congenital heart disease (CHD) and 30 healthy controls (HC) were enrolled in this study. Figure 1 shows the composition of the study cohort, characteristics are described in Table 1. Distribution of gender, gestational age and birth weight were similar. In the CHD group, less neonates were born by cesarean section (46.2%) compared to the control group (56.7%). Stool samples from CHD patients had been collected on day 7 of life, whereas samples from controls had been collected earlier most of the time (day 2 of life). The proportion of neonates who were exclusively breastfed did not differ between the two groups (62–67%). Nutrition of mothers during pregnancy was mainly meat containing. In the control group a larger proportion of mothers had received antibiotics during pregnancy (70 vs. 31%). The CHD group comprised 7 neonates with transposition of the great arteries (TGA), 4 with hypoplastic left heart syndrome (HLHS), and 1 with coarctation of the aorta and a common arterial trunk, respectively.

**Table 1:** General characteristics of the study population, including maternal factors.

	CHD (*n* = 13)	HC (*n* = 30)
Characteristics of neonates		
Female gender, *n* (%)	4 (30.8)	11 (36.7)
Fisher’s Exact Test on a 2 × 2 Contingency Table: *p*-value = 1		
Gestational Age, wk		
Mean ± SD	38.2 ± 2.7	39.0 ± 1.7
Median (minimum-maximum)	39.0 (33.0–41.3)	39.5 (33–41.4)
Welch Test (t test): *p*-value = 0.36		
Birth weight, g		
Mean ± SD	3153 ± 539	3332 ± 519
Median (minimum-maximum)	3350 (2100–3830)	3325 (2240–4360)
Welch Test (t test): *p*-value = 0.32		
Mode of delivery, *n* (%)		
Cesarean section	6 (46.2)	17 (56.7)
Vaginal delivery	7 (53.8)	13 (43.3)
Fisher’s Exact Test on a 2 × 2 Contingency Table: *p*-value = 0.74		
Age at first sampling, days		
Mean ± SD	7.8 ± 3.1	1.7 ± 1.5
Welch Test (t test): *p*-value <10^−4^		
Type of Feeding, *n* (%)		
Breastfeeding	8 (62)	20 (67)
Formula	3 (23)	10 (33)
Mixed	2 (15)	0 (0)
TPN	0	0 (0)
Chi-Squared Test: *p*-value = 0.08		
Major types of Congenital heart disease, *n*		
TGA	7	n.a.
COA	1	
HLHS	4	
TAC	1	
Characteristics of mothers		
Type of nutrition, *n* (%)		
meat containing	13 (100.0)	26 (86.7)
vegetarian	0 (0)	2 (6.7)
vegan	0 (0)	0 (0)
unknown	0 (0)	2 (6.7)
Chi-Squared Test: *p*-value = 0.38		
Antibiotic treatment during pregnancy, *n* (%)	4 (31)	21 (70)
Proportion Test: *p*-value = 0.04		
Known Group A *Streptococcus* colonization, n (%)	1 (8)	3 (10)
Proportion Test: *p*-value = 1		

Description of the general characteristics of neonates with congenital heart disease (CHD) and healthy controls (HC) as well as factors of the mothers potentially contributing to intestinal microbiota establishment and perturbation. Numbers (%), mean and standard deviation (SD) or median (minimum-maximum) are given for the different parameters as well as a comparison of the groups according to the suitable test. TGA, Transposition of the great arteries; COA, Aortic coarctation; HLHS, hypoplastic left heart syndrome; TAC, Common arterial trunk.

### 3.2 Differences in intestinal microbiota composition between CHD patients and HC with respect to mode of delivery

**Figure 2:**
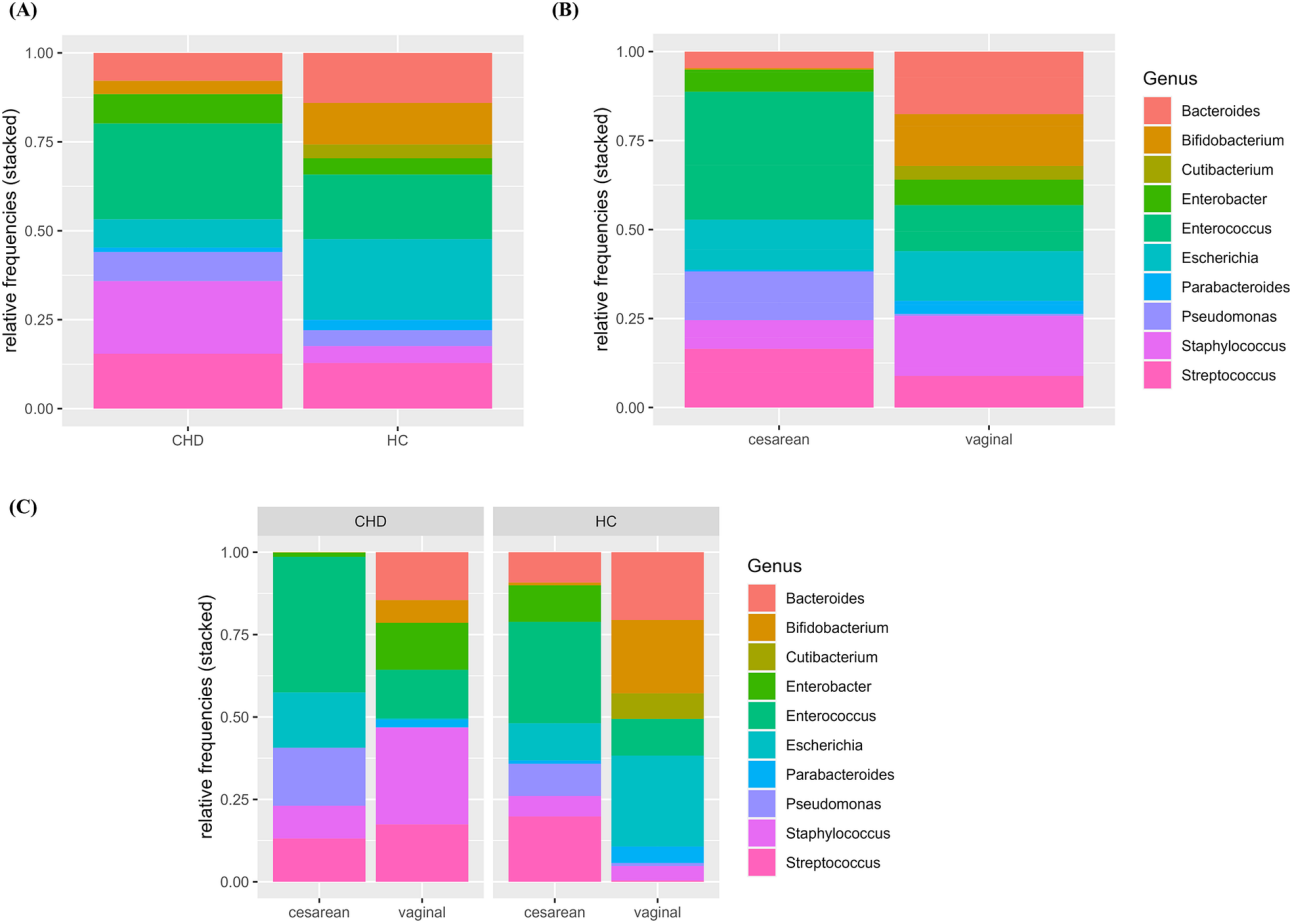
**(A–C)** Relative abundance of 10 most frequent genera by group and mode of delivery. Relative abundance (0–1.0) of the 10 most frequent genera are shown according to group **(A)**, mode of delivery **(B)** and the combination of group and mode of delivery **(C)**. HC, healthy control; CHD, congenital heart disease.

Composition and relative frequencies of the ten most abundant intestinal microorganisms in our study cohort were analyzed on the genus level (Figures 2A–C and Supplementary Table 2). The most abundant genera in the CHD group were *Enterococcus* (27%) and *Staphylococcus* (20%), whereas in HC fractions of *Escherichia* (23%) and *Enterococcus* (18%) were highest. Overall, CHD showed lower abundance of the genera *Bacteroides* (8% vs. 14%), *Parabacteroides* (1% vs. 3%), *Bifidobacterium* (4% vs. 12%), and *Escherichia* (8% vs. 23%). In contrast, the genera *Staphylococcus* (21% vs. 5%), *Enterococcus* (27% vs. 18%), *Enterobacter* (8% vs. 5%) and *Pseudomonas* (8% vs. 5%) were less prevalent in HCs (Figure 2A and Supplementary Table 2). Alpha diversity, reflected by Simpson’s, Shannon’s and InvSimpson indices was similar in both groups (Supplementary Figure 2A). Next, we analyzed the microbiota composition and abundance of the ten most frequent genera according to mode of delivery, but irrespective of disease state (Figure 2B). In neonates born by vaginal delivery the genera *Bacteroides, Bifidobacterium*, and *Staphylococcus* dominated, followed by *Escherichia* and *Enterococcus*. In contrast, neonates born by cesarean section presented a high proportion of the genus *Enterococcus*, and only a small fraction of *Bacteroides* and almost no *Bifidobacterium*. Of note, the genus *Pseudomonas* was only present in neonates born by cesarean section. Indices of alpha diversity were higher in neonates born by vaginal delivery (median HC (IQR)/median CHD (IQR)) with Shannon’s, Simpson’s and InvSimpson indices of 1.0 (IQR 0.66)/1.0 (IQR 0.88), 0.49 (IQR 0.19)/0.47 (IQR 0.42), 1.97 (IQR 1.05)/1.88 (IQR 1.79) versus 0.61 (IQR 0.31)/0.41 (IQUR 0.44), 0.32 (IQR 0.28)/0.19 (IQR 0.35) and 1.47 (IQR 0.72)/1.24 (IQR 0.73) in neonates born by cesarean section (median HC (IQR)/median CHD (IQR)), respectively (Supplementary Figure 2B and Supplementary Table 3).

**Figure 3:**
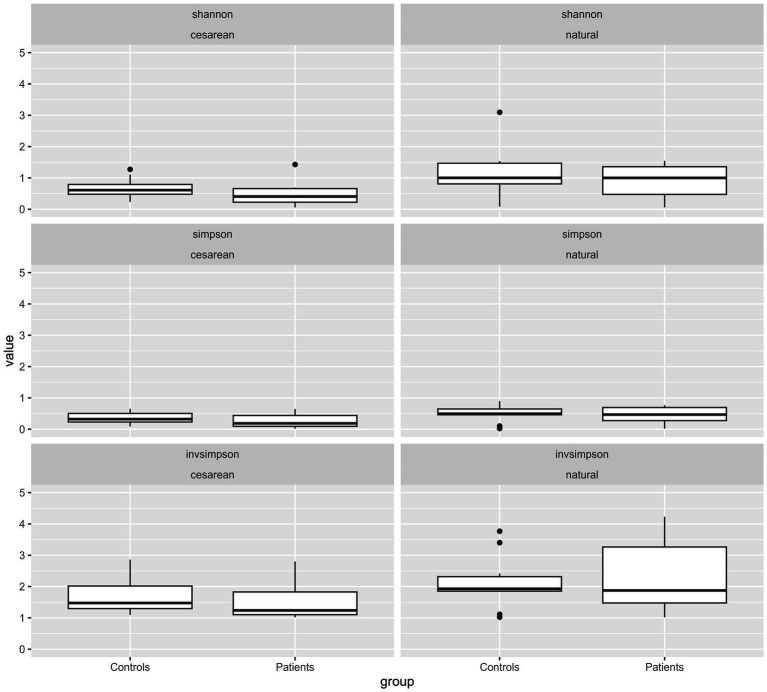
Diversities of all genera by type of birth according to patient cohort (CHD or HC). Box-Whisker Plots with median and interquartile range (IQR) of alpha diversity metrics (Shannon’s, Simpson’s and InvSimpson indices) of the intestinal microbiota composition according to mode of delivery and divided by patient group (CHD, HC). All values are displayed on the same scale to allow for comparison of different constellations. *p*-values for comparison are not given due to small numbers and lack of inference toward a general population.

To further explore differences in the microbiota composition between CHD patients and HCs, we analyzed composition and relative abundance on the genus level according to both, disease state as well as mode of delivery (Figure 2C and Supplementary Table 2B). Compared to their HCs, CHD patients that were born by vaginal delivery showed a lower fraction of the genera *Bacteroides* (15% vs. 21%) and *Bifidobacterium* (7% vs. 22%), and almost no *Escherichia* (0.4% vs. 28%). However, *Staphylococcus* (29% vs. 4%) and *Streptococcus* (17% vs. 0.4%) as well as *Enterobacter* (14% vs. 0.1%) were much more prevalent in CHD patients. In those CHD patients born by cesarean section, *Bacteroides* or *Bifidobacterium* was not found at all. Differences in the distribution of the other genera were not as pronounced between CHD patients and HC born by cesarean section, compared to those born by vaginal delivery. We could not detect obvious differences in alpha diversity indices within the four subgroups (Figure 3 and Supplementary Table 3).

### 3.3 Interplay between SpO2 and the intestinal core microbiota composition of infants with CHD

In order to detect a potential relationship between low oxygenation (SpO2 ≤ 90%) and altered perfusion parameters we analyzed intestinal oxygenation (O2C) and perfusion parameters in the total CHD cohort and two subcohorts (SpO2 ≤ 90% or > 90%) (Table 2). Blood flow, local hemoglobin amount, absolute oxygen saturation and blood flow velocity in the liver and periumbilical region tended to be lower in patients with SpO2 ≤ 90%. Results of the Doppler interrogation were highly variable and no clear trend in global gut perfusion/body weight and iFTOE could be observed. Total SpO2 correlated well with intestinal oxygenation (SO2 in the liver and periumbilical region) with adjusted R^2^ of 0.99 (SO2 in the liver) and 0.98 (SO2 in the periumbilical region) (Supplementary Figures 3A,B).

**Table 2:** Multivariate Analysis of Variance (MANOVA) on the effect of SpO2 on intestinal microbiota composition under consideration of mode of delivery.

Measure	Approximate F	*p*
SpO2	24.9	0.04
Mode of delivery	6.3	0.14
Interaction SpO2 and mode of delivery	3.1	0.27

Multivariate Analysis of Variance (MANOVA) reveals a significant effect of oxygen saturation measured by pulse oximetry (SpO2) on intestinal microbiota composition regardless of mode of delivery. Pillai’s trace was used. Degrees of freedom (Df) were 8 and 2.

**Figure 4:**
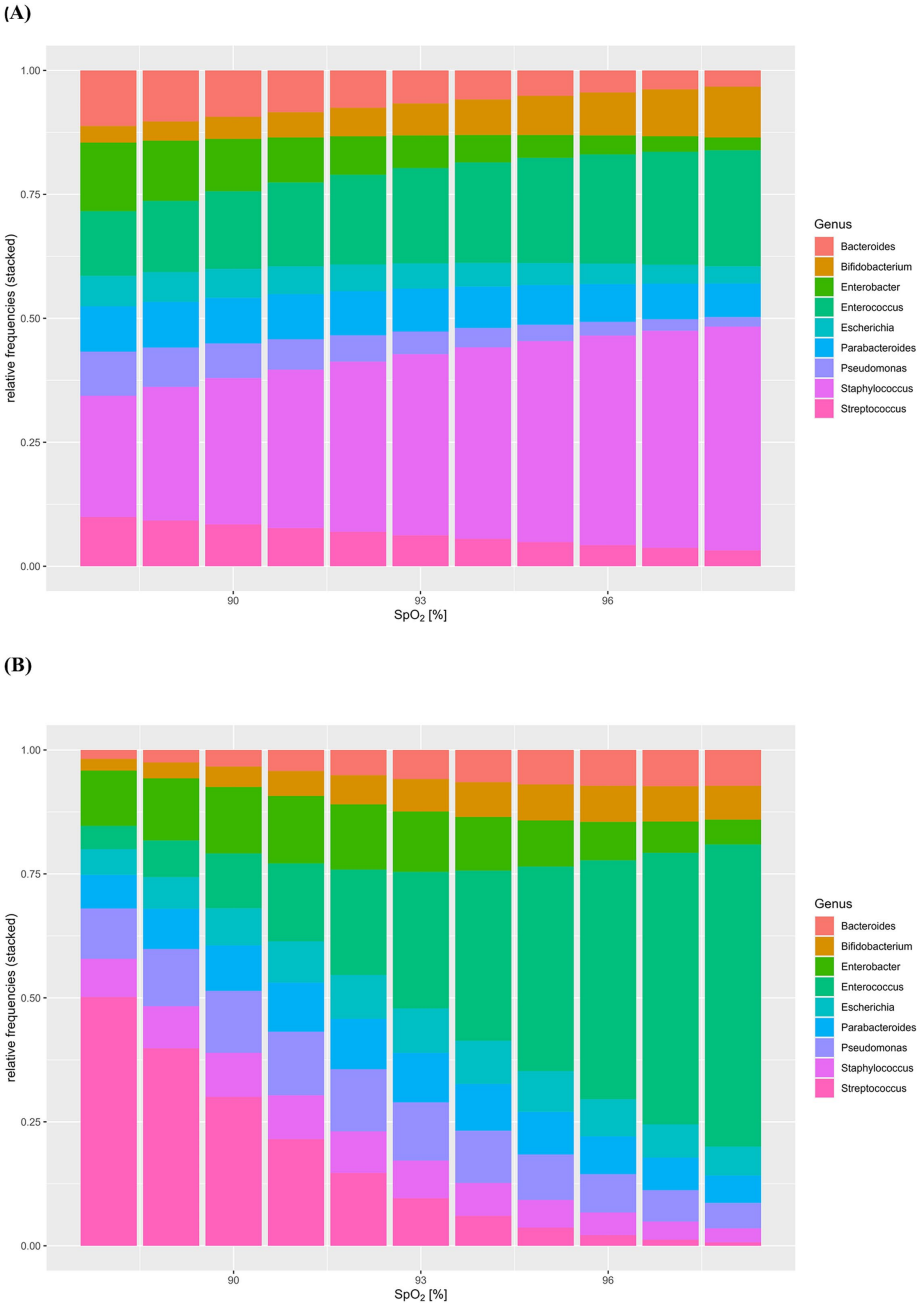
**(A,B)** Model of the effect of SpO2 on intestinal microbiota composition of congenital heart disease (CHD) patients under consideration of mode of delivery. Intestinal core microbiota composition data and oxygen saturation (SpO2) of neonates with CHD were used to create this visual, statistical model showing shifts in the intestinal core microbiota composition (*y*-axis: relative frequencies, 0–1.0) according to gradual alterations in SpO2 (%). Model for neonates with CHD born by vaginal delivery **(A)**, neonates with CHD born by cesarean section **(B)** was assessed by MANOVA.

Having determined the composition and abundance of the core microbiota of HC and CHD patients, we evaluated the effect of SpO2 on intestinal microbiota composition of CHD patients under consideration of the mode of delivery. MANOVA indicated a significant impact of SpO2 (*F*[8,2] = 24.9, *p* = 0.04) but not the mode of delivery (*F*[8,2] = 6.3, *p* = 0.14) on the intestinal microbiota composition of the 10 most frequent genera in neonates with CHD. Moreover, we did not find a significant interaction between SpO2 and mode of delivery (Table 3). The alteration of the core microbiota according to the potential SpO2 in neonates with CHD is illustrated by a model, shown in Figure 4 for patients born by vaginal delivery (Figure 4A and Supplementary Table 4A) and cesarean section (Figure 4B and Supplementary Table 4B). Overall, this visual, statistical model shows that proportional shifts in the intestinal core microbiota composition alongside gradual alterations in SpO2 are less pronounced in vaginal delivery compared to cesarean section. The model displays a large proportional shift from a microbiota dominated by the genus *Streptococcus* (50%) in conditions with low SpO2 toward a microbiota dominated by the genus *Enterococcus* (61%) in conditions with high SpO2. In addition, the proportions of the genera *Streptococcus, Pseudomonas* and *Enterobacter* are higher in conditions with low SpO2 compared to conditions with high SpO2. In contrast, proportions of the genera *Bifidobacterium* (2% with an SpO2 of 88% compared to 7% with an SpO2 of 98%) and *Enterococcus* (5% with an SpO2 of 88% compared to 61% with an SpO2 of 98%) increase with higher SpO2 in neonates born by cesarean section. Although less pronounced, this trend was also true for neonates born by vaginal delivery (Figures 4A,B and Supplementary Tables 4A,B).

**Table 3:** Oxygen-to-See (O2C) data and doppler interrogation for evaluation of oxygen metabolism and microperfusion, doppler-based resistive index and global gut perfusion data of neonates with SpO2 ≤ 90% and SpO2 > 90% and the total CHD cohort.

	CHD (SpO2 ≤ 90%) *N* = 7	CHD (SpO2 > 90%) *N* = 6	CHD (total cohort) *N* = 13
Mean SpO2 (%)	88.4 ± 2.3	93.7 ± 2.0	90.9 ± 3.4
Blood flow (Flow, AU)			
Liver (V. portae)	261.9 ± 42.8	278.4 ± 78.5	268.8 ± 57.6
Intestinal (periumbilical)	278.4 ± 46.2	297.2 ± 96.9	286.3 ± 68.4
Local hemoglobin amount (Hb, AU)			
Liver (V. portae)	63.0 ± 17.2	67.8 ± 6.3	65.0 ± 13.5
Intestinal (periumbilical)	58.0 ± 18.0	63.2 ± 18.7	60.2 ± 17.6
Absolute Oxygen Saturation (SO_2_, %)			
Liver (V. portae)	57.1 ± 7.2	59.6 ± 5.0	58.2 ± 6.2
Intestinal (periumbilical)	57.0 ± 9.3	61.4 ± 10.2	58.9 ± 9.5
Blood flow velocity (Velo, AU)			
Liver (V. portae)	66.1 ± 7.4	74.6 ± 13.8	69.7 ± 10.9
Intestinal (periumbilical)	73.1 ± 8.2	70.0 ± 8.1	71.8 ± 8.0
Doppler interrogation of superior mesenteric artery (SMA)			
SMA Peak systolic velocity (cm/s)	117.5 ± 57.4	98.0 ± 55.3	108.5 ± 55.0
SMA enddiastolic velocity (cm/s)	19.2 ± 8.7	12.9 ± 11.9	16.3 ± 10.4
SMA Resistance index	5.45 ± 12.2	6.3 ± 13.1	5.9 ± 12.1
SMA Pulsatility index	6.2 ± 11.8	7.3 ± 12.6.2	6.7 ± 11.7
Global gut perfusion (Portal vein volume blood flow, *Q*)			
Portal vein volume blood flow, *Q* per birth weight (ml/min)/kg	0.018 ± 0.002	0.018 ± 0.012	0.018 ± 0.007
Intestinal fractional oxygen extraction (iFTOE)			
iFTOE (%)	35.3 ± 8.8	36.2 ± 4.6	35.7 ± 7.1

Comparison of oxygenation and perfusion parameters in neonates with CHD according to oxygen saturation measured by pulse oximetry (SpO2) of ≤ 90 and > 90% with the entire CHD cohort. AU, area under the curve; Hb, hemoglobin; SO_2_, (local) oxygen saturation measured by the “oxygen to see” (O2C) method; SMA, superior mesenteric artery; Q, blood flow; iFTOE, intestinal fractional oxygen extraction. Groups were not statistically compared, since *p*-values would give hardly any clue toward generalization due to small numbers.

### 3.4 Hypothesis free cluster analysis

**Figure 5:**
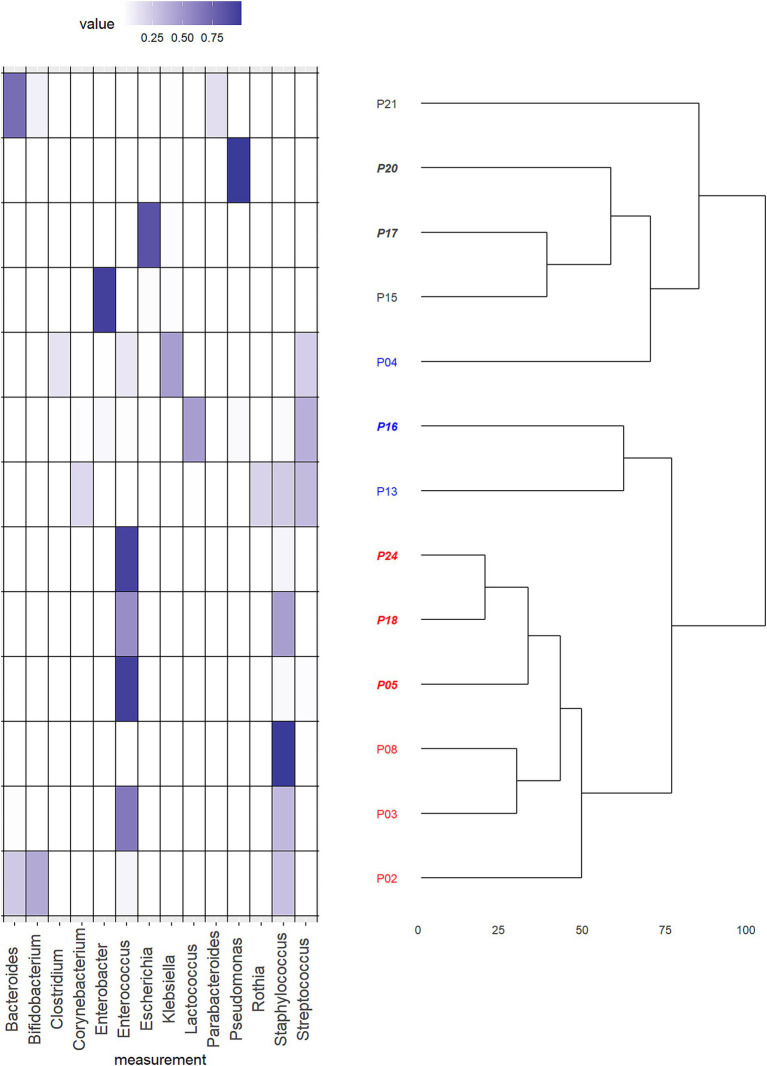
Hypothesis free cluster analysis of microbiota composition of patients with congenital heart disease (CHD). Heat map of relative abundances of the dominating genera in the total cohort of neonates with CHD (*n* = 13) in combination with the dendrogram resulting from an unsupervised hierarchical cluster analysis. Plotted genera were limited to those that resulted in a relative abundance of at least 0.1. The assignment of cases to final clusters followed essentially the results from the cluster analysis except case “P04.” This was reassigned to a different cluster due to its content of the genus *Streptococcus*. Patient numbers in *italic* are those born by cesarean section, others were born by vaginal delivery. Cluster 1 (red) was either dominated by the phylum Firmicutes and the genera *Staphylococcus* or *Enteroccocus* with low interindividual diversity. Cluster 2 (black) was mainly dominated by the phylum Proteobacteria, and the genera *Pseudomonas, Escherichia, Klebsiella* and *Enterobacter*, but showed high interindividual diversity. Cluster 3 (blue) consisted of 3 patients and was mainly characterized by a more diverse microbiota composition and presence of the genus *Streptococcus* in all patients. Other genera found in these patients were *Clostridium* and *Corynebacteria*, but no *Bacteroides* or *Bifidobacterium* at all.

To further delineate the differences in intestinal microbiota composition in an unbiased way, we performed an unsupervised clustering analysis of all bacterial species in CHD neonates (Figure 5). The analysis identified two main clusters (1-red and 2-black). Samples in Cluster 1 (red) were either dominated by the phylum Firmicutes and the genera *Staphylococcus* or *Enteroccocus* with low interindividual diversity. The microbiota of patients in Cluster 2 (black) was mainly dominated by the phylum Proteobacteria, and the genera *Pseudomonas, Escherichia, Klebsiella* and *Enterobacter*, but showed high interindividual diversity. However, by visual analysis of the heatmap (Figure 5), we identified a third sub-cluster which we then extracted. This Cluster 3 (blue) consisted of 3 patients only, but was mainly characterized by a more diverse microbiota composition and presence of the genus *Streptococcus* in all patients. Other genera found in these patients were *Clostridium* and *Corynebacteria*, but no *Bacteroides* or *Bifidobacterium* at all.

**Figure 6:**
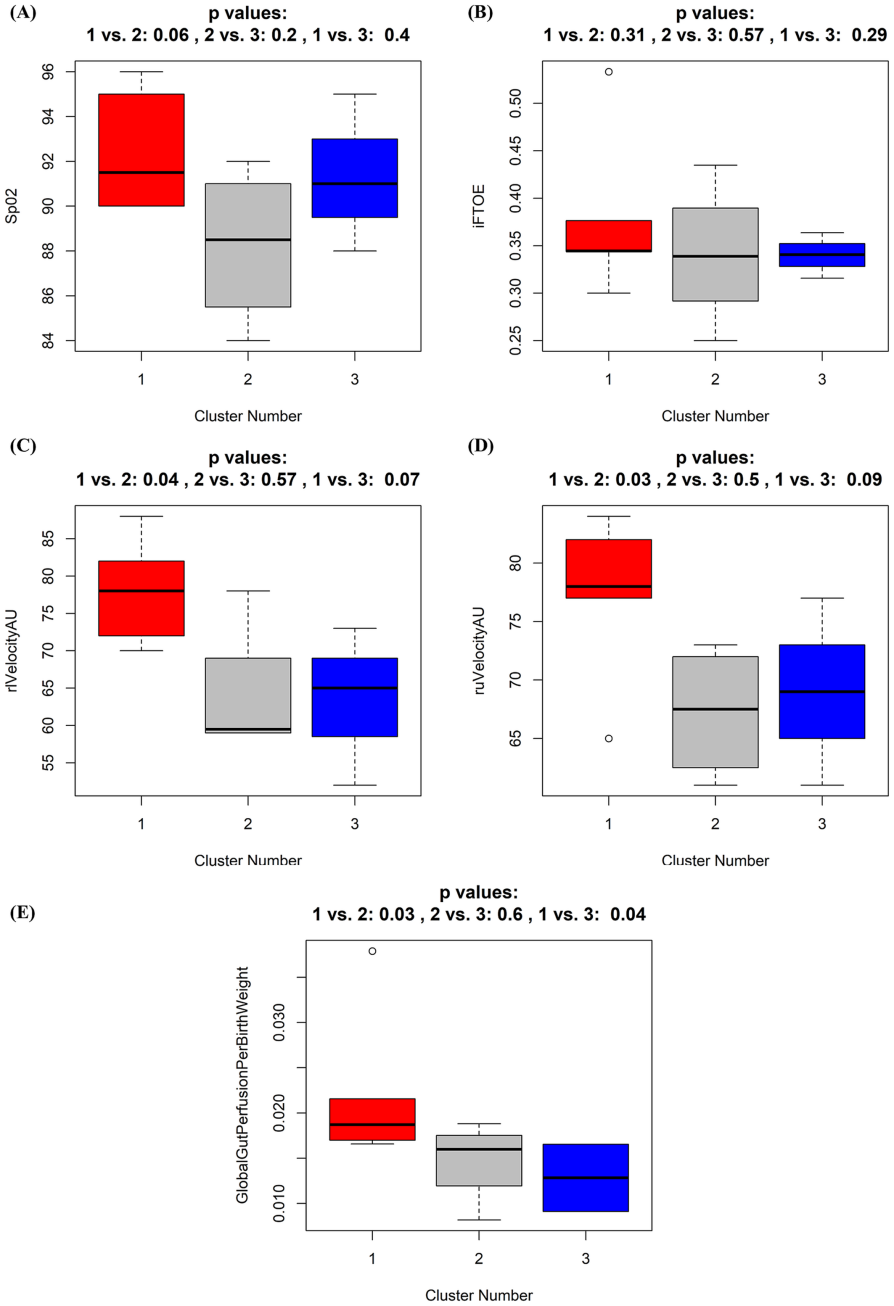
**(A–E)** Boxplots of oxygen saturation and perfusion parameters according to the three identified different microbiota composition clusters. Box whisker plots for oxygen saturation (SpO2, %) **(A)**, intestinal fractional tissue oxygen extraction (iFTOE, fraction) **(B)**, relative blood flow velocity in the liver (rlVelocityAU) **(C)** and umbilical region (ruVelocityAU) **(D)**, and global gut perfusion per birth weight **(E)** according to grouping by the three identified different microbiota composition clusters. Cluster 1 (red), Cluster 2 (black), Cluster 3 (blue). *p*-values result from Wilcoxon tests and applied oxygenation and perfusion parameters according to the three clusters. *p*-values here give rather the probability to see a more extreme result of the statistical test function within our sample subset than that actually proving the null hypothesis to be true. Thus, the result gives hardly any clue toward generalization.

In order to detect if similarities in overall microbiota composition, reflected by the Clusters 1–3 were associated with alterations of the intestinal perfusion and oxygenation, we compared overall SpO2, absolute oxygen saturation, blood flow, local hemoglobin amount, and blood flow velocity of the portal vein and periumbilical region, Resistance and Pulsatility Index of the superior mesenteric artery, global gut perfusion/kgKG and intestinal fractional tissue oxygen extraction (iFTOE) between the Clusters 1–3. No significant differences were found in absolute oxygen saturation, blood flow and local hemoglobin amount of the periumbilical region (Supplementary Figures 4A–C) or portal vein (Supplementary Figures 4D–F). Resistance and pulsatility index did not show any differences between clusters (Supplementary Figures 4G,H). Overall SpO2 and the iFTOE were similar between the Clusters (Figures 6A,B). However, perfusion parameters including blood flow velocity of the portal vein and periumbilical region as well as global gut perfusion related to body weight were significantly higher in Cluster 1 (Figures 6C–E).

## 4 Discussion

### 4.1 Early life intestinal core microbiota in neonates with CHD differs from HC

Investigating the composition and dynamics of the intestinal microbiome of neonates with CHD is essential to elucidate underlying mechanisms of early life gut microbiota perturbations and their impact on disease outcomes. Early life gut microbiome plays a critical role in maintaining host metabolic and immune homeostasis (Reynolds and Bettini, 2023; Zheng et al., 2020). Overall, we demonstrate a higher proportion of the phylum Firmicutes in neonates with CHD compared to HC. The genera *Staphylococcus, Enterococcus* and *Streptococcus* were the major contributors to the intestinal core microbiota composition in our entire cohort and were present in higher frequencies in neonates with CHD compared to HC. In contrast, the proportion of the genera *Bacteroides, Parabacteroides*, *Bifidobacterium* and *Escherichia* was decreased in CHD compared to HC. These alterations in microbiota composition in the first 2 weeks of life of CHD patients were similarly described in healthy neonates born by cesarean section (Penders et al., 2006; Azad et al., 2013; Biasucci et al., 2008; Dominguez-Bello et al., 2010; Rinninella et al., 2019). Thus, independent of mode of delivery, our data suggest similar alterations in the gut microbiota composition of neonates with CHD as seen in the C-section-associated disrupted maternal transmission of *Bacteroides* strains and dominance of healthcare-associated opportunistic pathogens, including *Enterococcus, Enterobacter* and *Klebsiella* species (Shao et al., 2019). Of note, less neonates with CHD (46%) were born by cesarean section compared to the HC group (57%) in our study.

By dissecting our cohort according to both, disease status and mode of delivery, our study identified a negative association of delivery by cesarean section and the abundance of the genera *Bacteroides* and *Bifidobacterium* irrespective of disease status, as already described by others (Penders et al., 2006; Bokulich et al., 2016; Reyman et al., 2019; Stewart et al., 2018). Furthermore, we could demonstrate that the depletion of the genera *Bacteroides* and *Bifidobacteria* was most pronounced in the CHD group born by cesarean section. Loss of *Bifidobacterium* species in early life is related to higher incidence of enteric inflammation and diseases such as necrotizing enterocolitis (NEC), systemic inflammation and sepsis in infants as well as with autoimmune disorders and atopic disease in later life (Arrieta et al., 2018; De Luca and Shoenfeld, 2019; Henrick et al., 2021; Masi and Stewart, 2019; Stewart et al., 2017). Additionally, the core microbiota of neonates in the CHD group who were born by cesarean section, was characterized by an overgrowth of the genera *Enterococcus* and *Pseudomonas*. First, this fits well to a recent finding by Huang et al., who mapped the early life gut microbiome in neonates with critical congenital heart disease and demonstrated an aberrant gut microbiome with an overgrowth of *Enterococcus* which was related to inflammatory response and poor prognosis (Huang et al., 2022). Authors hypothesized that the overgrowth of *Enterococcus* could mediate gut barrier impairment and reflect a crucial microbial feature for clinical prognosis in neonates with critical CHD. Second, a recent systematic review demonstrated that microbial dysbiosis preceding NEC in preterm infants is characterized by increased relative abundance of Proteobacteria and decreased abundance of Firmicutes and Bacteroidetes (Pammi et al., 2017). Relative frequencies of Firmicutes, in particular the genera *Staphylococcus* as well as *Bacteroides* were higher in neonates with CHD born by vaginal delivery in our study. Microbiome diversity did not differ according to disease status. However, in line with previous studies the comparison of gut bacterial *α*-diversity metrics was slightly higher in neonates born by vaginal delivery compared to cesarean section in both groups. Increased microbiota diversity in vaginally delivered neonates may be affected by the transmission of maternal gut microbiota, including non-colonizing microbiota (Kim et al., 2020; Lee et al., 2016).

In summary, our data show that the early life core microbiota of neonates with CHD differs from HC, independent of mode of delivery. However, disruption of the microbiota with a high proportion of Firmicutes, predominantly the genera *Enterococcus* and *Pseudomonas* alongside with a marked depletion of the genera *Bifidobacterium* and *Bacteroides* was pronounced in CHD born by cesarean section.

### 4.2 Core microbiota composition of neonates with CHD is influenced by SpO2

Several studies indicate a link between chronic hypoxia and alterations of the gut microbiome (Ma et al., 2023; Xing et al., 2018; Yan et al., 2024). Thus, we postulated that hypoxia and reduction of gut perfusion parameters secondary to poor cardiac output may influence composition of the early life core microbiota in neonates with CHD. Perfusion parameters in neonates with CHD and marked hypoxia (SpO2 ≤ 90%) did not largely differ from those with SpO2 > 90%. Of note, our study was not designed to find differences between both groups. We could also demonstrate a good correlation of the overall SpO2 in the total cohort with local tissue oxygenation in the liver and periumbilical region.

Multivariate Analysis of Variance revealed, that SpO2 significantly influences the intestinal core microbiota composition of patients with CHD, independent of the mode of delivery. Furthermore, in a model, we visualized the influence of altering levels of SpO2 on the relative frequencies of the intestinal core microbiota according to mode of delivery. Overall, the intestinal core microbiota seemed much more stable with respect to alterations in SpO2 in vaginal delivery compared to cesarean section. It seems possible, that the microbiota of vaginally delivered neonates with CHD is less affected by external influencing factors. Only recently, a comparison of the microbial profiles of 75 infants born vaginally or by planned versus emergent cesarean section suggested differences in colonization stability as an important factor in infant gut microbiome composition (Mitchell et al., 2020). Relative abundance of the genus *Bacteroides* between the first and second week of life was most stable in vaginally delivered neonates, whereas cesarean section was associated with loss of *Bacteroides* colonization. In line with this study, our cesarean section model revealed a marked depletion of the genera *Bifidobacterium* and *Bacteroides* alongside a dominance of Firmicutes (mainly genus *Streptococcus*) and an increase in the proportion of the genus *Pseudomonas* related to decreased oxygenation. On the contrary, we found that higher oxygen levels lead to a marked increase in Firmicutes/Bacteroidetes ratio, largely dominated by the genus *Enterococcus* after delivery by cesarean section and by the genera *Streptococcus* and *Enterococcus* after vaginal delivery.

In animal studies the impact of hyperoxia and oxygen inhalation has demonstrated a significant increase in the intestinal Firmicutes/Bacteroidetes ratio and a decrease in the relative frequencies of oxygen-intolerant microbes like Bacteroidetes and Lactobacillus in neonatal and adult mice (Cai et al., 2023; Li et al., 2021). Interestingly, the predominance of the genus *Enterococcus* increases markedly with better oxygenation levels in our model. Intestinal enterococcal translocation, potentially leading to subsequent systemic infections, has been associated with a certain threshold level of enterococcal overgrowth in the intestinal lumen in mice (Archambaud et al., 2019). Increased levels of intestinal fatty acid binding protein (FABP) and d-lactate as well as gelatinase secreted by *Enterococcus faecalis* have been associated with gut barrier dysfunction and stimulated inflammatory responses (Huang et al., 2022; Steck et al., 2011). In line with these findings, we hypothesize, that low oxygen levels may lead to a marked decrease of the beneficial genera *Bifidobacterium* and *Bacteroides* in parallel with a dominance of enteropathogens belonging to the genera *Pseudomonas* and *Streptococcus*. On the contrary, overgrowth of the genera *Enterococcus* and *Streptococcus* might occur in what we could call a “hyperoxic” state in CHD patients. Overall, our model demonstrates a possible impact of extremely low and extremely high oxygen levels on the intestinal core microbiota in neonates with CHD. However, alterations due to different oxygen levels seem less pronounced in neonates with CHD born by vaginal delivery, potentially due to a more stable intestinal microbiota composition.

### 4.3 Clusters of intestinal microbiota are associated with differences in global gut perfusion

To further identify naturally occurring differences in bacterial configurations between groups, we performed an unsupervised hierarchical clustering analysis of all microorganisms found in the samples of neonates with CHD at the genus level. The analysis, including a visually identified subgroup, mainly revealed three clusters dominated by either a dominance of a single genus belonging to the phylum Firmicutes (Cluster 1-red), the phylum Proteobacteria (Cluster 2-gray) or to a combination of Firmicutes (Streptococci) and Actinobacteria but with a total lack of the genera Bifidobacterium or Bacteroides (Cluster 3-blue).

Increasing evidence supports the concept of the gut-heart axis, including a link between heart failure and gut microbial dysbiosis (Almeida et al., 2023; Belli et al., 2023; Desai et al., 2023). Reduced systemic blood flow may alter the composition of the intestinal microbiota, which in turn can deteriorate the clinical course of heart failure (Formiga et al., 2019; Luedde et al., 2017; Matacchione et al., 2024). Considering this important hypothesis, it is plausible that similarly, CHD patients show alterations of key intestinal bacterial groups in situations with pulmonary hyperperfusion, drop of PVR and reduced systemic perfusion. Thus, we assessed in a second step how clusters were associated with oxygenation and perfusion parameters. For the first time, we could demonstrate a possible association of the intestinal microbiota composition in neonates with CHD with oxygenation and global gut perfusion. Although, not reaching statistical significance, neonates with an intestinal microbiota dominated by Proteobacteria (Cluster 2-gray) showed markedly lower oxygenation levels compared to the other two groups. Furthermore, compared to CHD neonates that showed an intestinal microbiota dominated by Firmicutes (genera Enterococcus and Staphylococcus, Cluster 1-red), global gut perfusion was significantly lower in those with a lack of the genera Bifidobacterium and Bacteroides (Cluster 3-blue). This phenomenon was also seen in those CHD neonates with a clear dominance of the phylum Proteobacteria (Cluster 2-gray).

In summary, our study provides evidence for a possible link between gut microbiota perturbations in neonates with CHD, oxygen supply and global gut perfusion. Our findings suggest that the early infant gut microbiota of CHD neonates lacks stability against external influencing factors like oxygen supply, especially in those born by cesarean section. Furthermore, we demonstrate that oxygenation (SpO2) significantly influences the intestinal core microbiota composition of neonates with CHD. Subgroups of patients with low global gut perfusion were characterized by either a dominance of pathobionts belonging to the phylum Proteobacteria or a high Firmicutes/Bacteroidetes ratio alongside a total absence of the beneficial genera *Bifidobacterium* or *Bacteroides* and a dominance of the genus *Streptococcus*. However, it remains unclear, whether these alterations in microbiota composition led to metabolomic perturbations and a subsequent excessive inflammatory response with impairment of the intestinal barrier in our patient cohort. Another plausible explanation is, that hypoperfusion, reflected by impaired global gut perfusion itself leads to intestinal barrier dysfunction and subsequently facilitates bacterial translocation of dominating pathobionts especially in those individuals with a high Firmicutes/Bacteroidetes ratio or an overgrowth of Proteobacteria (Bischoff et al., 2014).

We acknowledge several limitations of our findings and their evaluation. Firstly, this study was mainly observational and therefore, associations mentioned cannot be seen as proof of causation. Secondly, the sample size of our patient cohort may be considered small and can limit generalizability of our findings. Thirdly, due to the nature of our cohort composition, samples from healthy neonates were collected earlier (day 2) in comparison to samples from neonates with CHD (day 7). Therefore, we cannot exclude that intergroup differences observed between HC and CHD are solely related to disease status, as others have shown slightly differing abundance of bacterial communities with repeated assessments of fecal microbiota at day 3 and day 7 of life (Kim et al., 2020; Combellick et al., 2018). Other factors, which may influence gut microbiota such as nasogastric tube feeding practice and the influence on SpO2 from other sources of oxygen such as endotracheal tube leak during invasive ventilation inflating the intestine, have not been considered in our study. However, due to our approach we could exclude that HC collected their samples outside the hospital environment at home, which we anticipated to have a bigger impact on the composition due to the current literature (Combellick et al., 2018; Selma-Royo et al., 2024). Other factors, including nutritional ones did not largely differ between groups. Last, we did not analyze longitudinal data of our patient cohort within this publication, which could give a clearer picture on the long-term development of the intestinal microbiota in neonates with CHD with regards to influences of the PICU environment and the impact of altered oxygenation and perfusion during and after cardiopulmonary bypass surgery. Further, longitudinal studies with a larger sample size and an analysis of the functional activity of microbial populations are needed to elucidate the stochasticity of our observations as well as their biological and clinical impact.

Overall, our study highlights differences between the intestinal core microbiota composition of neonates with CHD compared to healthy controls that are present shortly after birth. Our study suggests that alterations in the core microbiota composition might be caused by different oxygenation levels of neonates with CHD. However, in line with findings from recent studies, we hypothesize that mode of delivery could be responsible for its overall stability in early life. Distinct patterns with dominance of potential gram-negative pathogens and a lack of beneficial Bifidobacterium species might be associated with a reduction in global gut perfusion. Our results provide important evidence that pharmaceutical interventions like probiotic administration or fecal transplant could be an interesting investigational approach for neonates with CHD in order to restore the natural balance of the intestinal microbiota, prevent intestinal pathobionts from overgrowth and systemic translocation and subsequently lower the rate of nosocomial infections.

## Data Availability

The datasets presented in this study can be found in online repositories. The names of the repository/repositories and accession number(s) can be found in the article/Supplementary material.

## Footnotes

^1^ https://www.ebi.ac.uk/ena/browser/home

^2^ https://github.com/UliSchopp/IMIC
