# Supplementary material for: Oxygenation and intestinal perfusion and its association with perturbations of the early life gut microbiota composition of children with congenital heart disease
Source: Front Microbiol. 2025 Jan 15;15:1468842. doi: 10.3389/fmicb.2024.1468842 (PMC11775010; doi:10.3389/fmicb.2024.1468842)
Supplement: Supplementary file 1 [file Data_Sheet_1.docx]

Supplementary Material

1. **Supplementary Methods**

# 1 Bar Plots

Figures 2A, 2B and 2C were made by

- Selection the appropriate data for patients at visit 1 and for controls
- Matching the information about way of delivery
- Normalization of absolute abundancies to get relative ones
- Calculation of means of relative abundancies for each genus
- Selection of the largest ten groups
- Renormalization after selection

## R Scripts

Following R scripts were used

- CompositionBarPlots_1a.R
- CompositionBarPlots_1b.R

## Packages used

| dplyr | 1.1.3 |
| --- | --- |
| readxl | 1.4.3 |
| ggplot2 | 3.5.0 |
| tidyr | 1.3.0 |

## References

[1] R Core Team (2023). *R: A Language and Environment for Statistical Computing*. R Foundation for Statistical Computing, Vienna, Austria. <https://www.R-project.org/>.

[2] Wickham H, François R, Henry L, Müller K, Vaughan D (2023). *dplyr: A Grammar of Data Manipulation.* R package version 1.1.3, <https://CRAN.R-project.org/package=dplyr>.

[3] Wickham H, Bryan J (2023). *readxl: Read Excel Files.* R package version 1.4.3, <https://CRAN.R-project.org/package=readxl>

[4] H. Wickham. *ggplot2: Elegant Graphics for Data Analysis.* Springer-Verlag New York, 2016.

[5] Wickham H, Vaughan D, Girlich M (2023). tidyr: Tidy Messy Data. R package version 1.3.0, <https://CRAN.R-project.org/package=tidyr>.

# Diversities

The following diversity measures were calculated

1. Shannon index
2. Simpson index
3. Inverted Simpson index

for the genera data grouped either

1. by controls and patients

or by

1. by controls and patients and way of delivery

Essentially the *vegan* package was employed (version 2.6.4). To prepare the data routines from the packages *dplyr* (version 1.1.3), *readxl* (version 1.4.3) and *Hmisc* (version 5.1.1) were used. The plots were made with *ggplot2* (version 3.5.0) and data tables written by *writexl* (version 1.4.2).

## Data Preparation

The bulk of work was to select patient’s data on gene abundance for the first visit only and to merge with information about way of delivery. Gene abundances were normalized in the common way, so that the abundances for every case summed up to one (relative abundances).

## R Scripts

Two R script were written. The code is not very complex.

- F_Diversity_Healtyh_CHD_zuV1_Version_03.R
  - Supplementary Figures 1A
- F_Diversity_Healtyh_CHD_zuV1_VagCaesar.R
  - Supplementary Figures 1B, 1C
  - Table 3

## References

[1] R Core Team (2023). *R: A Language and Environment for Statistical Computing*. R Foundation for Statistical Computing, Vienna, Austria. <https://www.R-project.org/>.

[2] Wickham H, François R, Henry L, Müller K, Vaughan D (2023). *dplyr: A Grammar of Data Manipulation.* R package version 1.1.3, <https://CRAN.R-project.org/package=dplyr>.

[3] Wickham H, Bryan J (2023). *readxl: Read Excel Files.* R package version 1.4.3, <https://CRAN.R-project.org/package=readxl>

[4] Oksanen J, Simpson G, Blanchet F, Kindt R, Legendre P, Minchin P, O'Hara R, Solymos P, Stevens M, Szoecs E, Wagner H, Barbour M, Bedward M, Bolker B, Borcard D, Carvalho G, Chirico M, De Caceres M, Durand S, Evangelista H, FitzJohn R, Friendly M, Furneaux B, Hannigan G, Hill M, Lahti L, McGlinn D, Ouellette M, Ribeiro Cunha E, Smith T, Stier A, Ter Braak C, Weedon J (2022). *vegan: Community Ecology Package.* R package version 2.6-4, <https://CRAN.R-project.org/package=vegan>.

[5] H. Wickham. *ggplot2: Elegant Graphics for Data Analysis.* Springer-Verlag New York, 2016.

[6] Harrell Jr F (2023). *Hmisc: Harrell Miscellaneous*. R package version 5.1-1, <https://CRAN.R-project.org/package=Hmisc>.

[7] Ooms J (2023). *writexl: Export Data Frames to Excel 'xlsx' Format*. R package version 1.4.2, <https://CRAN.R-project.org/package=writexl>.

# Linear Models

It would be interesting to establish a formula that models the relative bacterial composition as a function of the amount of oxygenation (SpO_2_) and of method of delivery:

$Y_{k,l} \sim SPO2_{k}+ MOD_{k}$ (1)

with

- $Y_{k,l}$ the relative bacterial composition for case *k* and the bacterial genus *l*
- $SPO2_{k}$ the amount of oxygenation for case *k*
- $MOD_{k}$ the mode of delivery for case *k*.

Several things must be mentioned here:

1. We write model formulas in the convention used in R
   1. $Y\sim X$ just indicates, that there exists a generic relation between *Y* and *X*.
   2. $Y\sim X+U$ is then meant to express, that the relation is somehow linear in *X* and *U*. In fact the equation behind that is something like

$Y_{k,l}= \alpha_{l}\times SPO2_{k}+\beta_{l}\times MOD_{k}+\varepsilon_{k,l}$ (2)

with coefficients $\alpha_{l},\beta_{l}$ that are to be estimated from the data and $\varepsilon_{k,l}$ being the error term, which obeys some assumed distribution. In linear regression, this assumed error distribution is often taken as the standard normal distribution.

1. The relative compositions $Y_{k,l}$ are not suited for linear regression analysis, bat its isometric log-transforms are! Instead of formula (1) we apply

${ilr(Y}_{k,l}) \sim SPO2_{k}+ MOD_{k}$ (3)

- The mode of delivery is modeled by a dummy variable, that is by 0 or 1, respectively. Model assessment is performed by an ANOVA.
- One may include some interaction effects between oxygenation and delivery. We observed that including that some of the coefficient are more significantly different from zero. But as we have only few data and interpretation of models with interaction is difficult, we abandoned that route.
- Using the estimated model (2) it is possible to predict potential compositions from amount of oxygenation and mode of delivery as inputs. Results were plotted in two separate figures. The range of oxygenation should not exceed that one in the sampled data to provide for extrapolation effect.

1. We had to restrict the number of genera to nine due to the amount of data.

## Steps Performing the Model estimation

1. We selected the patients’ data at the first visit.
2. After a first provisional normalization we calculated the mean value for each genus and selected the nine largest ones.
3. This was followed by a second normalization now taking only the nine genera into account.
4. Data were merged with information about oxygenation and about way of delivery.
5. Compositional data were ilr-transformed.
6. Linear regression was performed.
7. ANOVA was employed.
8. Composition was predicted for a range of oxygenation resulting in ilr-transformed data. This was retransformed to relative abundances.

## R Script

We used code in a R Markup file “AuswertungFuerPaper_LinareModelle_9_Genera_spO2AsContinous_Delivery_PlotCompositions_FarbenAngepasst”.

## Packages used

| dplyr | 1.1.3 |
| --- | --- |
| dtplyr | 1.3.1 |
| readxl | 1.4.3 |
| compositions | 2.0.6 |
| cluster | 2.1.4 |
| ggplot2 | 3.5.0 |
| ggdendro | 0.1.23 |
| tidyr | 1.3.0 |
| grid | 4.3.1 |
| scales | 1.3.0 |

## References

[1] R Core Team (2023). *R: A Language and Environment for Statistical Computing*. R Foundation for Statistical Computing, Vienna, Austria. <https://www.R-project.org/>.

[2] Wickham H, François R, Henry L, Müller K, Vaughan D (2023). *dplyr: A Grammar of Data Manipulation.* R package version 1.1.3, <https://CRAN.R-project.org/package=dplyr>.

[3] Wickham H, Bryan J (2023). *readxl: Read Excel Files.* R package version 1.4.3, <https://CRAN.R-project.org/package=readxl>

[4] H. Wickham. *ggplot2: Elegant Graphics for Data Analysis.* Springer-Verlag New York, 2016.

[5] Wickham H, Girlich M, Fairbanks M, Dickerson R (2023). dtplyr: Data Table Back-End for 'dplyr'. R package version 1.3.1, <https://CRAN.R-project.org/package=dtplyr>.

[6] van den Boogaart KG, Tolosana-Delgado R, Bren M (2023). compositions: Compositional Data Analysis. R package version 2.0-6, <https://CRAN.R-project.org/package=compositions>.

[7] Maechler, M., Rousseeuw, P., Struyf, A., Hubert, M., Hornik, K. (2022). cluster: Cluster Analysis Basics and Extensions. R package version 2.1.4.

[8] de Vries A, Ripley BD (2022). ggdendro: Create Dendrograms and Tree Diagrams Using 'ggplot2'. R package version 0.1.23, <https://CRAN.R-project.org/package=ggdendro>.

[9] Wickham H, Vaughan D, Girlich M (2023). tidyr: Tidy Messy Data. R package version 1.3.0, <https://CRAN.R-project.org/package=tidyr>.

[10] Wickham H, Pedersen T, Seidel D (2023). scales: Scale Functions for Visualization. R package version 1.3.0, <https://CRAN.R-project.org/package=scales>.

# Cluster Analysis

We wanted to get an impression about relevance of relative abundances of genera for a separation of patients at first visit. For that we performed the following steps:

1. Filtering: Data of absolute abundances were read from tables and filtered to get the data for the patients at the day of first visit.
2. Imputation: Any zeros occurring in the absolute abundances were substituted by 0.001.
3. Normalization: Absolute abundances were then normalized to get relative abundances.
4. Cluster Analysis:
   1. Isometric log-ration transformation (called ilr transformation in short) was performed applying the ilr routine from the *compositions* package.
   2. Cluster analysis was performed on the ilr transformed data by the agnes routine coded in the *cluster* package. Parameter used were
      1. metric = "euclidean"
      2. method = “ward”
5. Heat Map: Routines from the packages *ggdendro*, *ggplot2* and *grid* were employed to get heat maps of relative abundances were we limited the plotted genera to 14: Those that resulted in a relative abundance of at least 0.1.

-
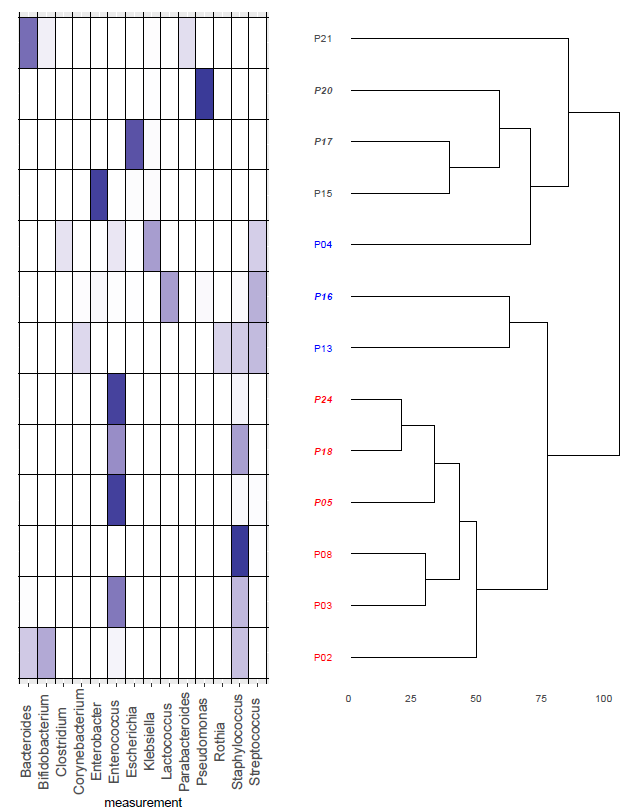


Figure 1: Dendrogram combined with heatmap

1. The heatmap and the dendrogram resulting from the cluster analysis were combined.
2. The assignment of cases to final clusters followed essentially the results from the cluster analysis except case “P04”. This was reassigned to a different cluster due its content of *streptococcus* (cf Figure 1).
3. Finally box whisker plots were created for several clinical parameters like SpO_2_. These plots were grouped by three cluster identified in step 5.

-
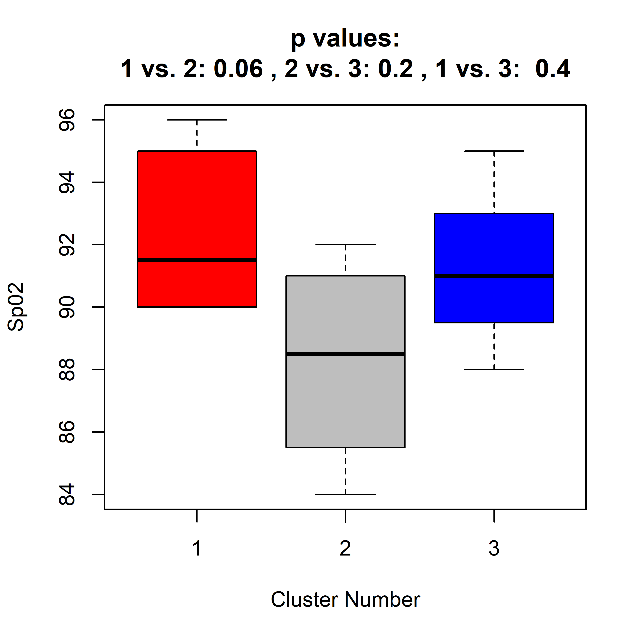


Figure 2: Example of a box whisker plot

Added to the plots were p-values resulting from Wilcoxon tests applied clinical parameter according to the three cluster. We want to remind here, that p-values give the probability to see a more extreme result of the statistical test function than that actually seen for the samples given the null hypothesis to be true. So they stick to the samples and give hardly any clue towards generalization. As the sample sized are rather small one should not ponder them to heavily.

## R Scripts

Three R scripts were written. As the code is a bit complex we preferred to use R Markdown files. Descriptions are in German. But the steps should be reproducible by anyone with some knowledge in R.

- Auswertung_BWPlots_DreiClusterParameterSet_2_Farbig.Rmd
- Auswertung_BWPlots_GlobalGut_Farbig.Rmd
- Auswertung_BWPlots_SpO2_Farbig.Rmd

The code differs only in the data used for the box whisker plots. To integrate all in one seemed too complicated.

## Packages used

| dplyr | 1.1.3 |
| --- | --- |
| dtplyr | 1.3.1 |
| readxl | 1.4.3 |
| compositions | 2.0.6 |
| cluster | 2.1.4 |
| ggplot2 | 3.5.0 |
| ggdendro | 0.1.23 |
| tidyr | 1.3.0 |
| grid | 4.3.1 |

## References

[1] R Core Team (2023). *R: A Language and Environment for Statistical Computing*. R Foundation for Statistical Computing, Vienna, Austria. <https://www.R-project.org/>.

[2] Wickham H, François R, Henry L, Müller K, Vaughan D (2023). *dplyr: A Grammar of Data Manipulation.* R package version 1.1.3, <https://CRAN.R-project.org/package=dplyr>.

[3] Wickham H, Bryan J (2023). *readxl: Read Excel Files.* R package version 1.4.3, <https://CRAN.R-project.org/package=readxl>

[4] H. Wickham. *ggplot2: Elegant Graphics for Data Analysis.* Springer-Verlag New York, 2016.

[5] Wickham H, Girlich M, Fairbanks M, Dickerson R (2023). dtplyr: Data Table Back-End for 'dplyr'. R package version 1.3.1, <https://CRAN.R-project.org/package=dtplyr>.

[6] van den Boogaart KG, Tolosana-Delgado R, Bren M (2023). compositions: Compositional Data Analysis. R package version 2.0-6, <https://CRAN.R-project.org/package=compositions>.

[7] Maechler, M., Rousseeuw, P., Struyf, A., Hubert, M., Hornik, K. (2022). cluster: Cluster Analysis Basics and Extensions. R package version 2.1.4.

[8] de Vries A, Ripley BD (2022). ggdendro: Create Dendrograms and Tree Diagrams Using 'ggplot2'. R package version 0.1.23, <https://CRAN.R-project.org/package=ggdendro>.

[9] Wickham H, Vaughan D, Girlich M (2023). tidyr: Tidy Messy Data. R package version 1.3.0, <https://CRAN.R-project.org/package=tidyr>.

**Supplementary Tables**

**Table 1: Tools and sources used for the bioinformatic analysis**

| **tool** | **version** | **download link** | **doi / paper** |
| --- | --- | --- | --- |
| fastp | v0.20.1 | <https://github.com/OpenGene/fastp> | 10.1093/bioinformatics/bty560 |
| kraken2 | v2.0.9beta | <https://ccb.jhu.edu/software/kraken2/> | 10.1186/s13059-019-1891-0 |
| kneaddata | v0.10.0 | <https://github.com/biobakery/kneaddata> |  |
| trimmomatic | v0.39 | <https://github.com/usadellab/Trimmomatic> | 10.1093/bioinformatics/btu170 |
| bowtie2 | v2.4.5 | <https://github.com/BenLangmead/bowtie2> | 10.1038/nmeth.1923 |
| diamond | v2.0.13 | <https://github.com/bbuchfink/diamond> | 10.1038/nmeth.3176 |
| Megan | v6.20.19 | <https://uni-tuebingen.de/fakultaeten/mathematisch-naturwissenschaftliche-fakultaet/fachbereiche/informatik/lehrstuehle/algorithms-in-bioinformatics/software/megan6/> | 10.1101/gr.5969107 |

**Table 2A: Relative frequencies of the ten most abundant genera according to patient group**

| **Genus** | **HC (n=30)** | **CHD (n=13)** |
| --- | --- | --- |
| Bacteroides | 14,06% | 7,83% |
| Bifidobacterium | 11,71% | 3,72% |
| Cutibacterium | 3,85% | 0,06% |
| Enterobacter | 4,60% | 8,25% |
| Enterococcus | 18,13% | 26,93% |
| Escherichia | 22,70% | 7,96% |
| Parabacteroides | 2,92% | 1,26% |
| Pseudomonas | 4,47% | 8,17% |
| Staphylococcus | 4,74% | 20,41% |
| Streptococcus | 12,83% | 15,43% |

**Legend:** Relative frequencies of the ten most abundant genera in percentage (%). Values are displayed in the main manuscript as Figure 2A. HC – healthy controls; CHD – congenital heart disease.

**Table 2B: Relative frequencies of the ten most abundant genera according to mode of delivery**

| **Group** | **Genus** | **Vaginal delivery (n=20)** | **Cesarean**  **Section**  **(n=23)** | **p-value** |
| --- | --- | --- | --- | --- |
| HC | Bacteroides | 20,57% | 9,22% | **0.015*** |
| CHD | Bacteroides | 14,53% | 0,00% | 0.257 |
| **p-value** |  | 0.197 | 0.921 |  |
| HC | Bifidobacterium | 22,26% | 0,82% | **0.003**** |
| CHD | Bifidobacterium | 6,90% | 0,01% | 0.337 |
| **p-value** |  | **0.08** | 0.921 |  |
| HC | Cutibacterium | 7,71% | 0,00% | 0.112 |
| CHD | Cutibacterium | 0,00% | 0,13% | 0.355 |
| **p-value** |  | 0.163 | 0.276 |  |
| HC | Enterobacter | 0,09% | 11,09% | 1.0 |
| CHD | Enterobacter | 14,24% | 1,25% | 0.699 |
| **p-value** |  | 0.731 | 0.866 |  |
| HC | Enterococcus | 11,09% | 30,77% | 0.934 |
| CHD | Enterococcus | 14,72% | 41,17% | 0.329 |
| **p-value** |  | 0.921 | 0.494 |  |
| HC | Escherichia | 27,58% | 11,28% | **0.016*** |
| CHD | Escherichia | 0,40% | 16,78% | 0.93 |
| **p-value** |  | **0.004**** | 1.0 |  |
| HC | Parabacteroides | 5,01% | 1,01% | **0.025**** |
| CHD | Parabacteroides | 2,33% | 0,00% | 0.44 |
| **p-value** |  | **0.066** | 0.496 |  |
| HC | Pseudomonas | 0,93% | 9,78% | 0.514 |
| CHD | Pseudomonas | 0,08% | 17,60% | 0.385 |
| **p-value** |  | 0.534 | 0.354 |  |
| HC | Staphylococcus | 4,39% | 6,20% | 0.75 |
| CHD | Staphylococcus | 29,39% | 9,92% | 0.713 |
| **p-value** |  | 0.351 | 0.576 |  |
| HC | Streptococcus | 0,36% | 19,82% | 0.183 |
| CHD | Streptococcus | 17,39% | 13,14% | 0.698 |
| **p-value** |  | 0.296 | 0.482 |  |

**Legend:** Relative frequencies of the ten most abundant genera in percentage (%) according to mode of delivery. Values are displayed in the main manuscript as Figure 2B and 2C. Data were grouped by one of two distinct properties (either by mode of delivery or by CHD/HC) conditional on the second group. That is for example patients (CHD) were compared to healthy controls (HC) conditional on vaginal mode of delivery. A Wilcoxon test was applied to the abundances of genera. HC – healthy controls; CHD – congenital heart disease. Cesarean section (n=23 includes 6 neonates with CHD and 17 HC), vaginal delivery (n=20 includes 7 neonates with CHD and 13 HC).

**Table 3: Alpha diversity metrics of the intestinal microbiota composition according to patient group and mode of delivery.**

| **Group** | **Mode of delivery** | **Index** | **Median** | **IQR** |
| --- | --- | --- | --- | --- |
| HC | cesarean | shannon | 0,61 | 0,31 |
| CHD | cesarean | shannon | 0,41 | 0,44 |
| HC | vaginal | shannon | 1,00 | 0,66 |
| CHD | vaginal | shannon | 1,00 | 0,88 |
| HC | cesarean | simpson | 0,32 | 0,28 |
| CHD | cesarean | simpson | 0,19 | 0,35 |
| HC | vaginal | simpson | 0,49 | 0,19 |
| CHD | vaginal | simpson | 0,47 | 0,42 |
| HC | cesarean | invsimpson | 1,47 | 0,72 |
| CHD | cesarean | invsimpson | 1,24 | 0,73 |
| HC | vaginal | invsimpson | 1,97 | 1,05 |
| CHD | vaginal | invsimpson | 1,88 | 1,79 |

**Legend:** Median and interquartile range (IQR) of alpha diversity metrics of the intestinal microbiota composition of neonates with congenital heart disease (CHD, n=13) and healthy controls (HC, n=30) are shown.

**Table 4A: Modelled proportional shift in the intestinal core microbiota composition according to gradual alteration in oxygen saturation (SpO2) in neonates with CHD born by vaginal delivery.**

| **SpO2** | **Bacteroides** | **Bifidobacterium** | **Enterobacter** | **Enterococcus** | **Escherichia** | **Parabacteroides** | **Pseudomonas** | **Staphylococcus** | **Streptococcus** |
| --- | --- | --- | --- | --- | --- | --- | --- | --- | --- |
| 88 | 11,2% | 3,3% | 13,8% | 13,0% | 6,1% | 9,1% | 8,9% | 24,4% | 9,9% |
| 89 | 10,3% | 3,9% | 12,1% | 14,4% | 6,0% | 9,2% | 7,9% | 27,0% | 9,2% |
| 90 | 9,4% | 4,5% | 10,5% | 15,6% | 5,8% | 9,2% | 7,0% | 29,5% | 8,5% |
| 91 | 8,4% | 5,1% | 9,1% | 16,9% | 5,6% | 9,1% | 6,1% | 32,0% | 7,7% |
| 92 | 7,5% | 5,8% | 7,8% | 18,1% | 5,4% | 8,9% | 5,3% | 34,3% | 6,9% |
| 93 | 6,7% | 6,4% | 6,6% | 19,2% | 5,1% | 8,7% | 4,6% | 36,6% | 6,2% |
| 94 | 5,9% | 7,2% | 5,5% | 20,3% | 4,8% | 8,4% | 3,9% | 38,6% | 5,5% |
| 95 | 5,1% | 7,9% | 4,6% | 21,2% | 4,5% | 8,0% | 3,3% | 40,5% | 4,9% |
| 96 | 4,4% | 8,7% | 3,8% | 22,0% | 4,1% | 7,6% | 2,8% | 42,3% | 4,3% |
| 97 | 3,8% | 9,5% | 3,1% | 22,8% | 3,8% | 7,2% | 2,3% | 43,8% | 3,7% |
| 98 | 3,3% | 10,3% | 2,6% | 23,4% | 3,5% | 6,8% | 1,9% | 45,1% | 3,2% |

**Legend:** Model of relative frequencies (percentage, %) of the intestinal core microbiota composition of neonates with CHD born by vaginal delivery according to gradual alterations in SpO2. SpO2 – oxygen saturation measured by pulse oximetry. Values are visualized as Figure 3A in the main manuscript.

**Table 4B: Modelled proportional shift in the intestinal core microbiota composition according to gradual alteration in oxygen saturation (SpO2) in neonates with CHD born by vaginal cesarean section.**

| **SpO2** | **Bacteroides** | **Bifidobacterium** | **Enterobacter** | **Enterococcus** | **Escherichia** | **Parabacteroides** | **Pseudomonas** | **Staphylococcus** | **Streptococcus** |
| --- | --- | --- | --- | --- | --- | --- | --- | --- | --- |
| 88 | 1,79% | 2,37% | 11,15% | 4,69% | 5,19% | 6,79% | 10,15% | 7,69% | 50,18% |
| 89 | 2,51% | 3,21% | 12,54% | 7,38% | 6,40% | 8,10% | 11,55% | 8,48% | 39,82% |
| 90 | 3,34% | 4,13% | 13,40% | 11,04% | 7,49% | 9,19% | 12,48% | 8,89% | 30,03% |
| 91 | 4,22% | 5,05% | 13,61% | 15,70% | 8,34% | 9,91% | 12,81% | 8,85% | 21,52% |
| 92 | 5,09% | 5,88% | 13,16% | 21,26% | 8,84% | 10,17% | 12,53% | 8,39% | 14,68% |
| 93 | 5,86% | 6,56% | 12,18% | 27,53% | 8,97% | 9,98% | 11,73% | 7,61% | 9,59% |
| 94 | 6,49% | 7,02% | 10,83% | 34,29% | 8,74% | 9,43% | 10,55% | 6,64% | 6,02% |
| 95 | 6,95% | 7,26% | 9,31% | 41,25% | 8,23% | 8,60% | 9,17% | 5,59% | 3,65% |
| 96 | 7,21% | 7,29% | 7,76% | 48,15% | 7,53% | 7,61% | 7,73% | 4,57% | 2,15% |
| 97 | 7,30% | 7,14% | 6,31% | 54,77% | 6,70% | 6,56% | 6,35% | 3,64% | 1,23% |
| 98 | 7,22% | 6,83% | 5,01% | 60,93% | 5,84% | 5,53% | 5,11% | 2,84% | 0,69% |

**Legend:** Model of relative frequencies (percentage, %) of the intestinal core microbiota composition of neonates with CHD born by cesarean section according to gradual alterations in SpO2. SpO2 – oxygen saturation measured by pulse oximetry. Values are visualized as Figure 3B in the main manuscript.

## Supplementary Figures

**Figure 1: Median relative abundance and interquartile range of bacterial genera found in Microbial Community Standard Samples alongside every extraction process (n=12).**

**
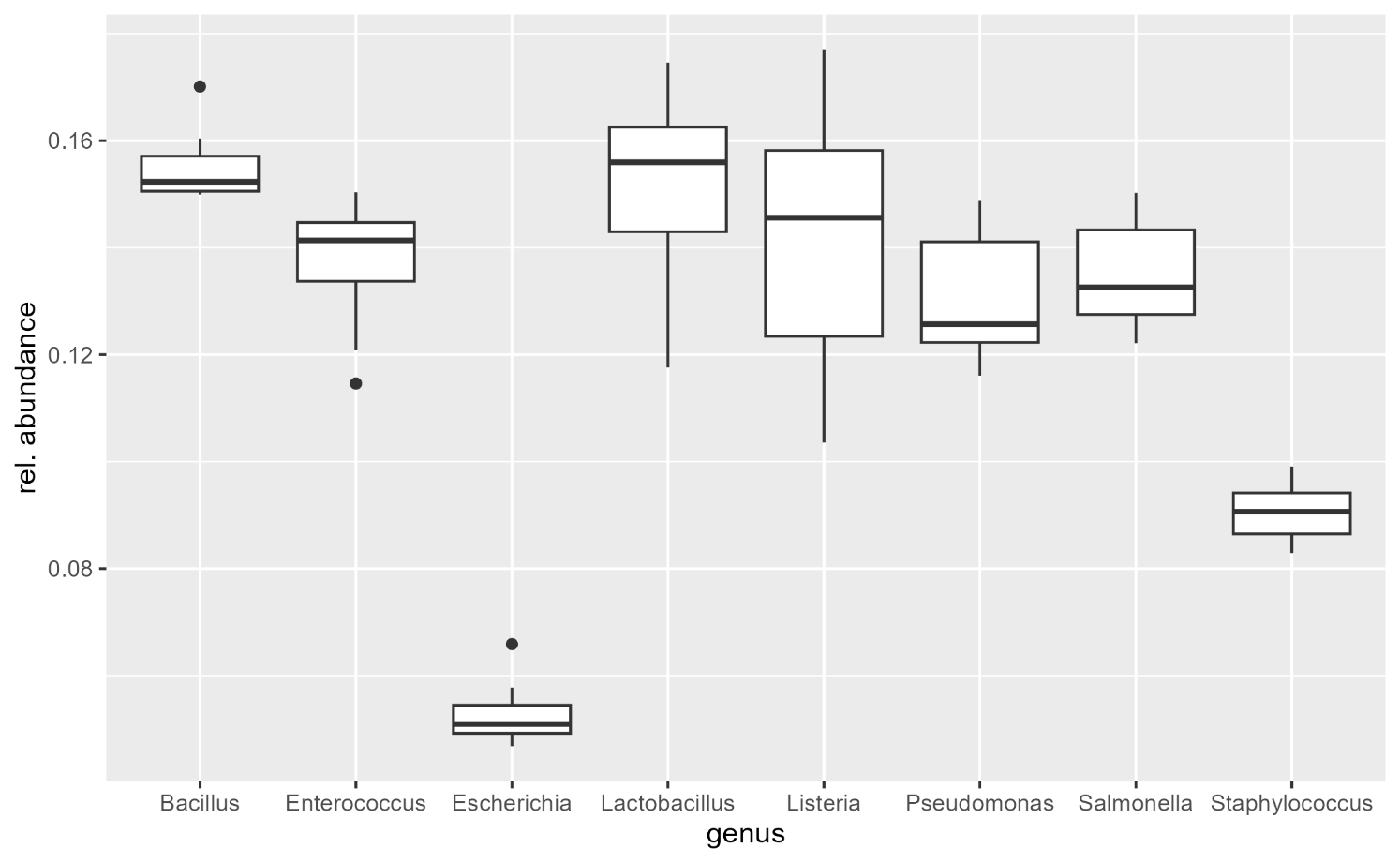
**

**Legend:** Box-Whisker Plots of relative abundance of the microbiota composition of microbial community standard samples (MOC samples) as a control alongside every extraction process are shown. X-Axis shows different bacterial genera detected, Y-Axis shows relative abundance. Values are median and interquartile range (IQR). The expected distribution was a relative abundance of 12.5% of each species.

**Figure 2A: Diversities of all genera for HC and CHD.**


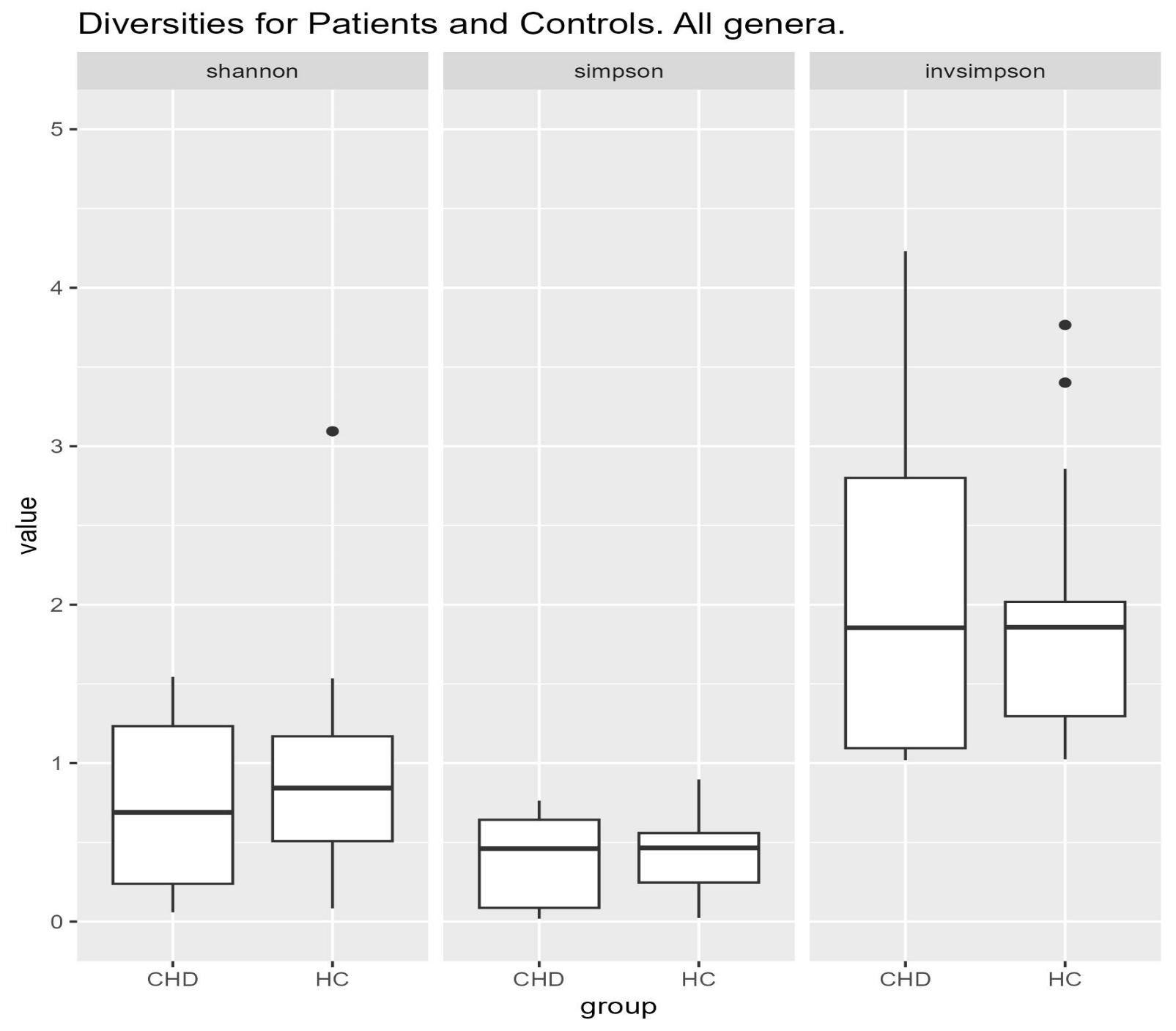


**Legend:** Box-Whisker Plots with median and interquartile range (IQR) of alpha diversity metrics (Shannon’s, Simpson’s and InvSimpson indices) of the intestinal microbiota composition of neonates with congenital heart disease (CHD, n=13) and healthy controls (HC, n=30) are shown and are similar between groups. P-values for comparison are not given due to small numbers and lack of inference towards a general population.

**Figure 2B: Diversities of all genera by type of birth.**
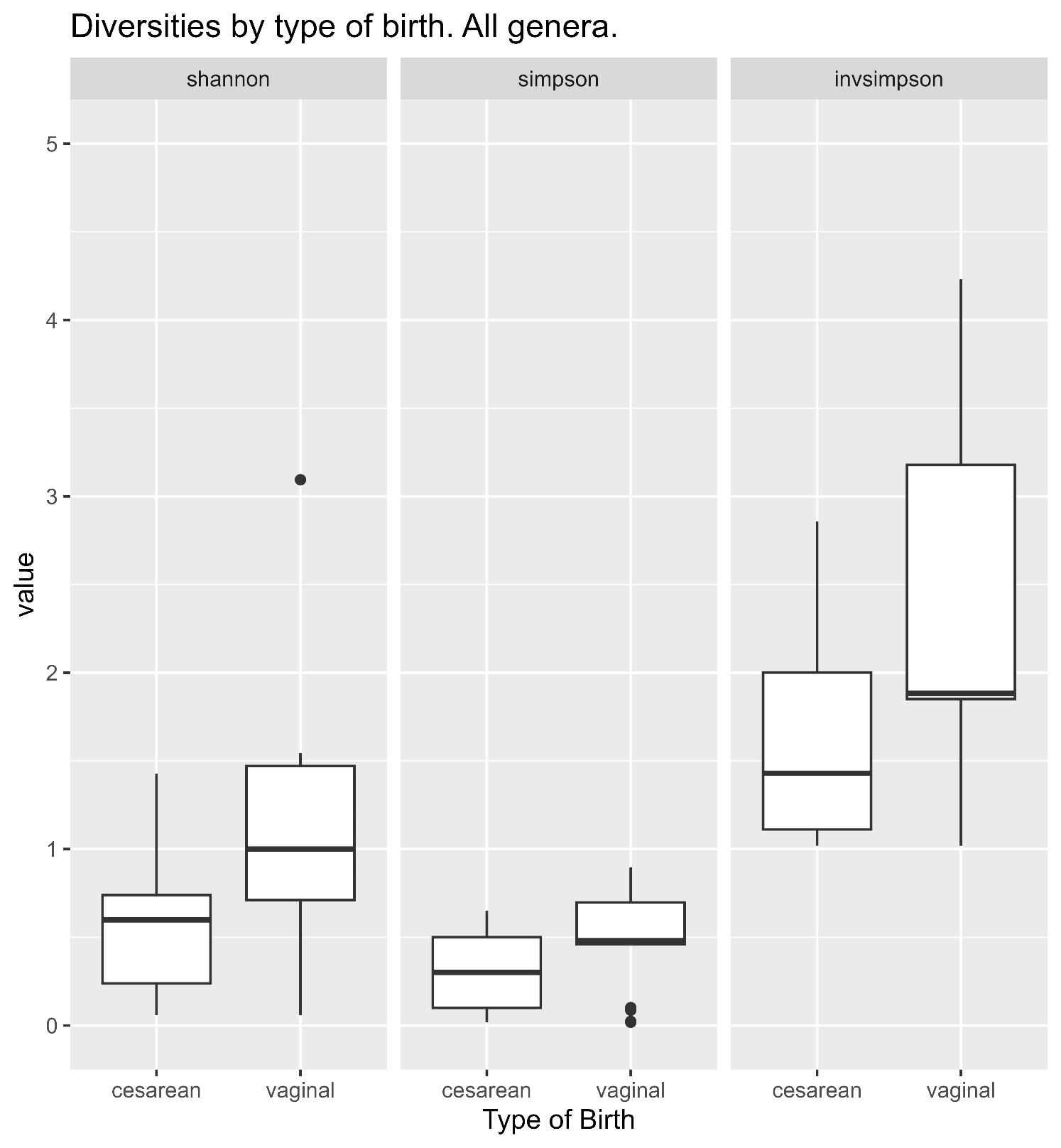


**Legend:** Box-Whisker Plots with median and interquartile range (IQR) of alpha diversity metrics (Shannon’s, Simpson’s and InvSimpson indices) of the intestinal microbiota composition according to mode of delivery. Alpha diversity metrics were slightly higher in vaginally born neonates. P-values for comparison are not given due to small numbers and lack of inference towards a general population.

**Figure 3A: Linear Model, correlation of SpO2 – rl SO2.**

Adjusted R² 0.9879, Estimate 0.64


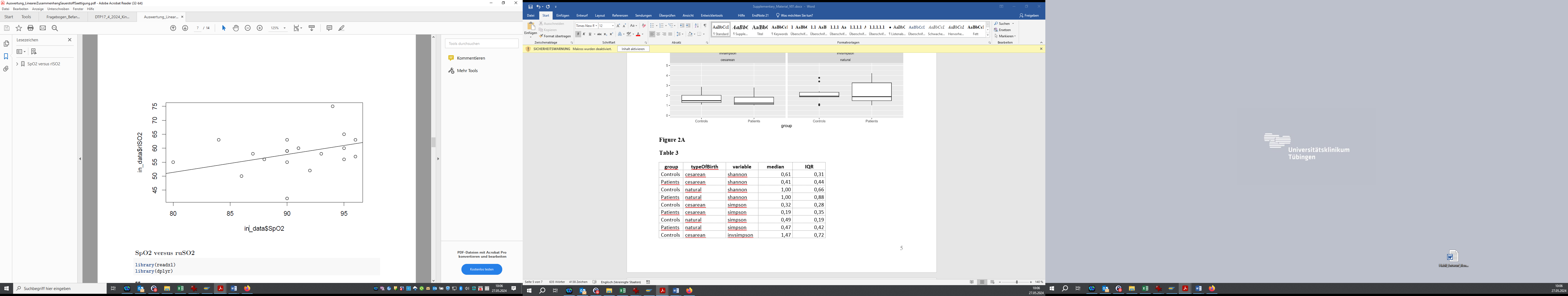


**Figure 3B: Linear Model, correlation of SpO2 – ru SO2**

Adjusted R² 0.9765, Estimate 0.66


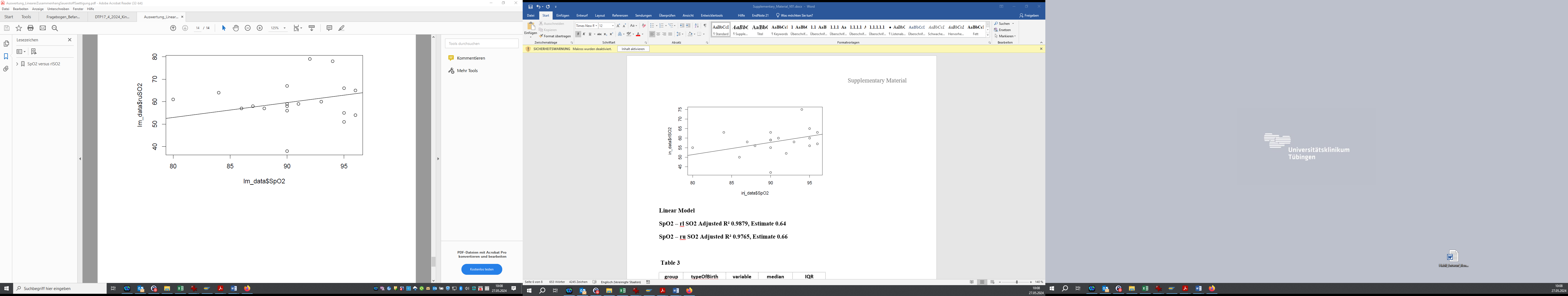


For correlation of SpO2 and rlSO2/ruSO2 all measurements in our patient cohort performed were used (n=17 for rlSO2 and n=18 for ruSO2).

**Figure 4: Comparison of intestinal oxygenation and perfusion parameters according to clusters of intestinal microbiota composition in patients with CHD (n=13)**

**(A)**

**
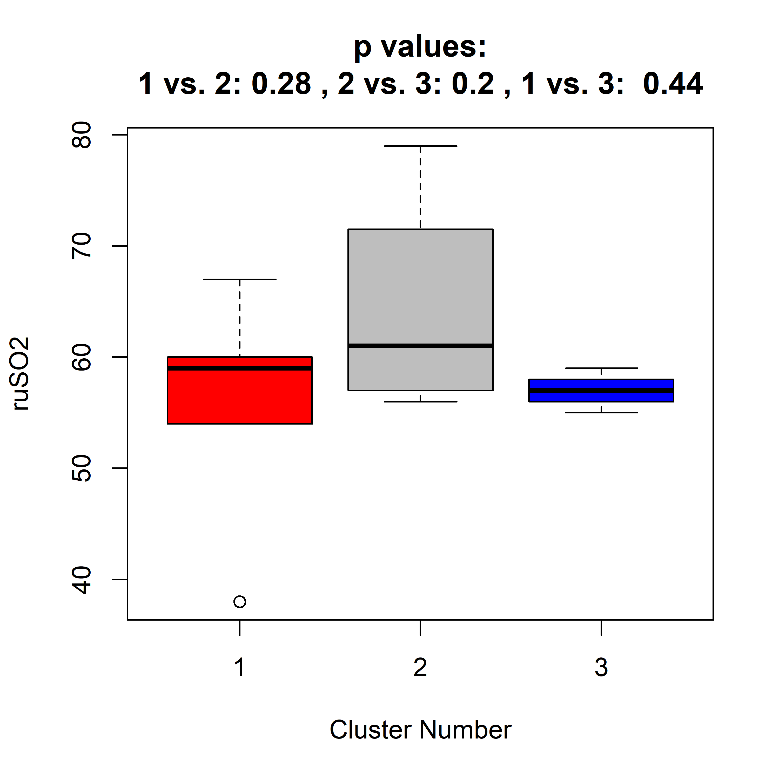
**

**(B)**

**(C)
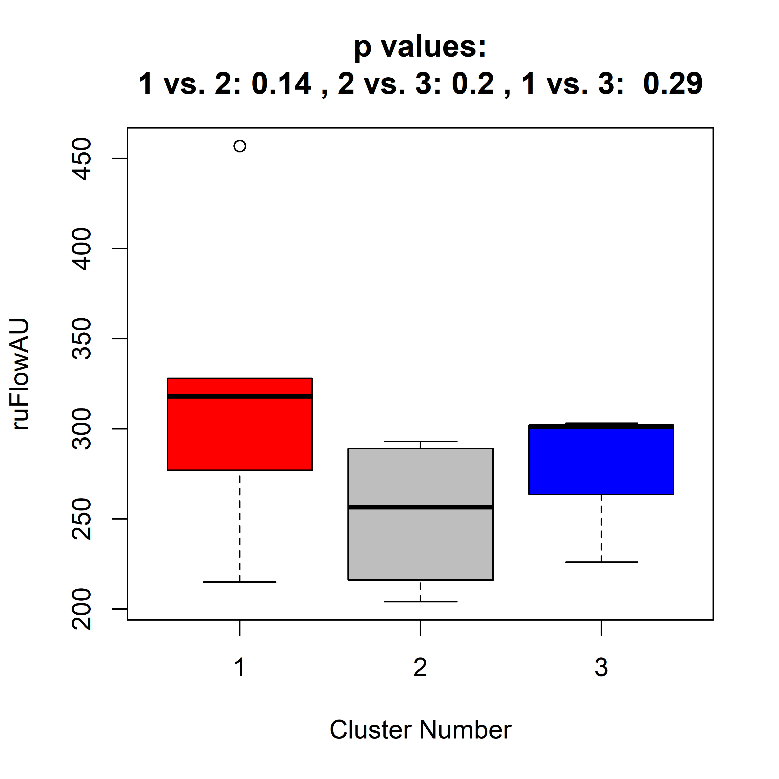

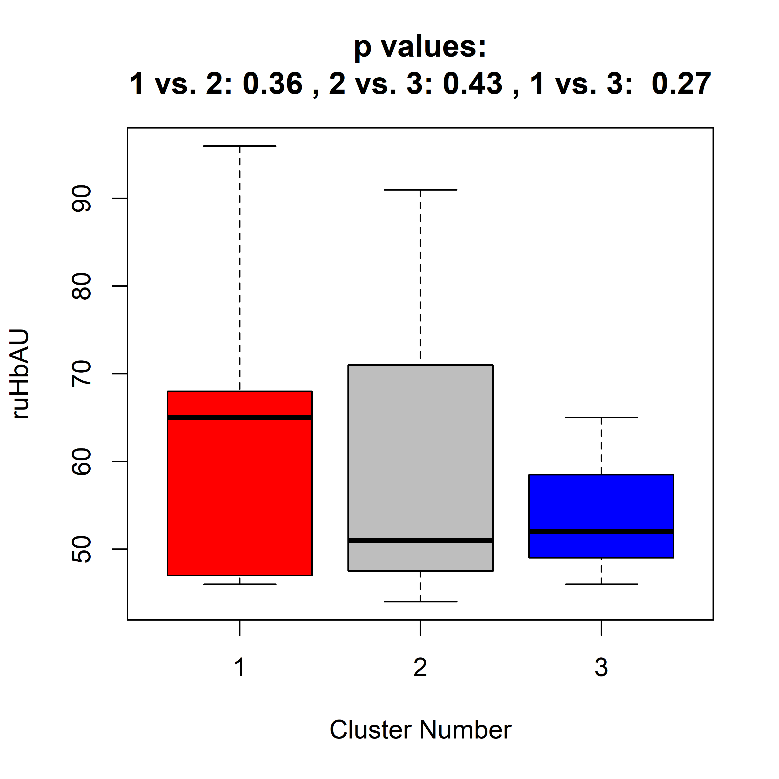
**

**(D)**

**
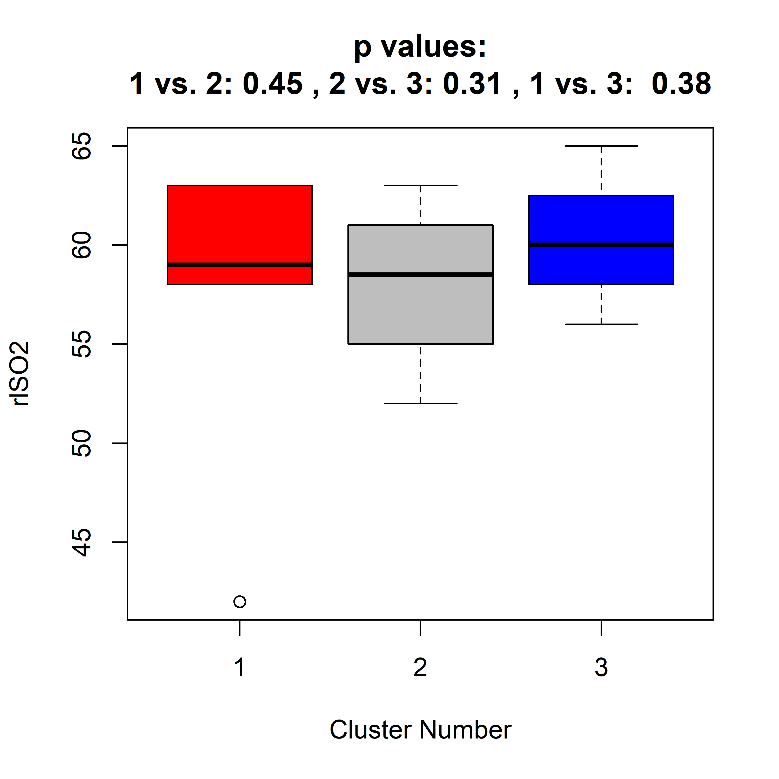
**

**(E)**

**
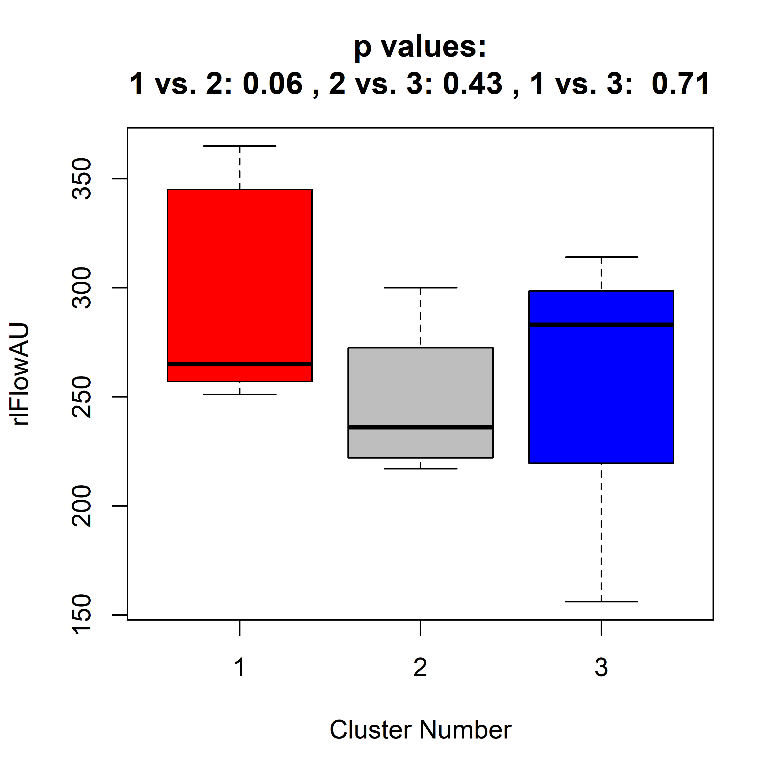
**

**(F)**

**
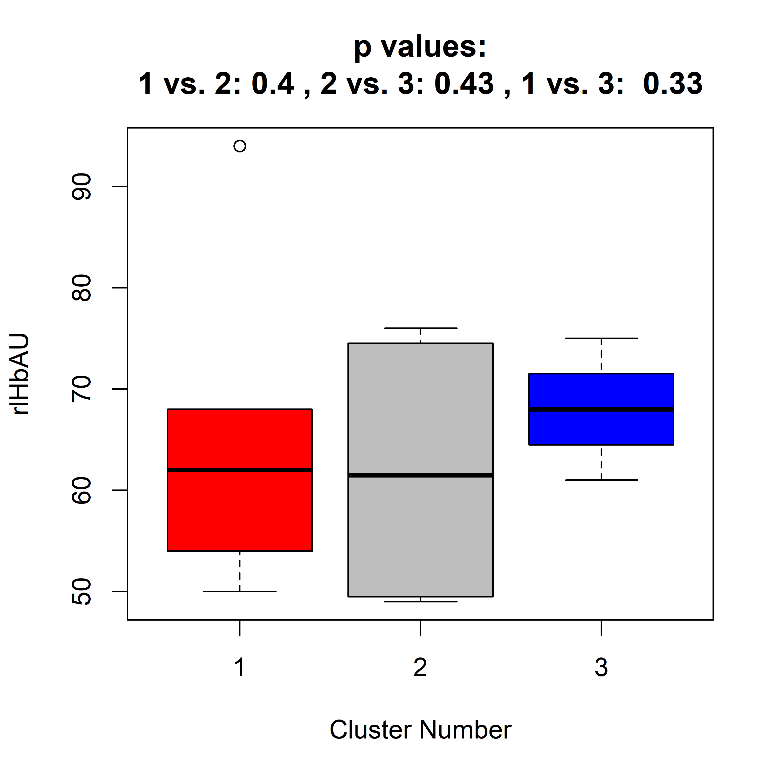
**

**(G)**

**
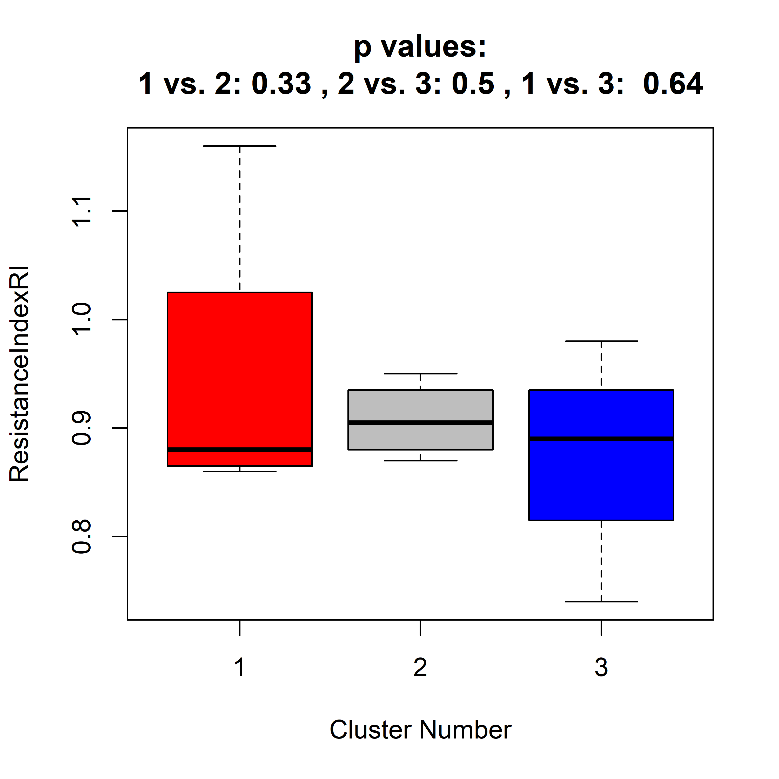
**

**(H)**

**
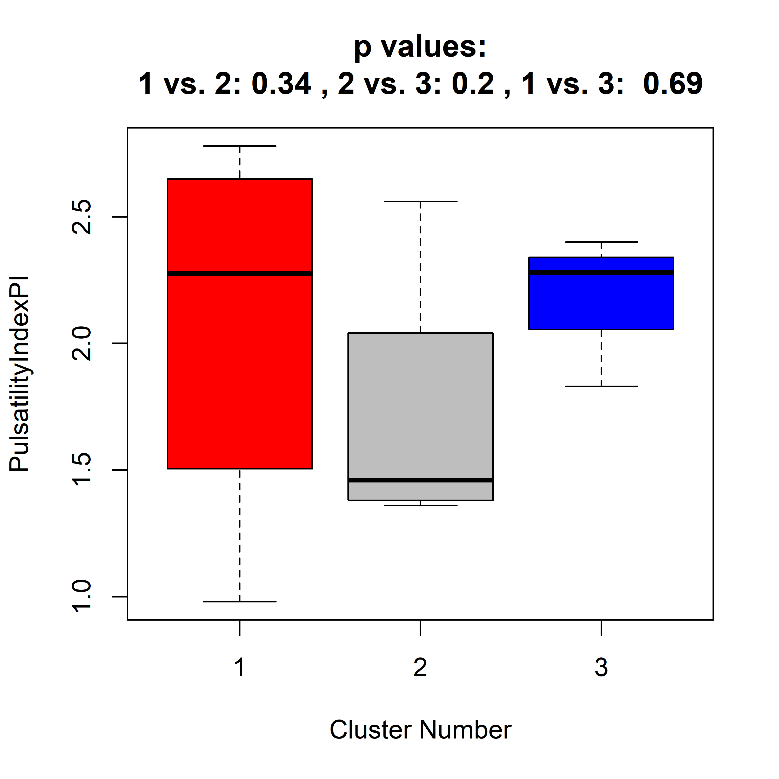
**

**Legend:** Comparison of intestinal oxygenation and perfusion parameters according to clusters of intestinal microbiota composition in patients with CHD (n=13) by Box-Whisker Plots with median and interquartile range (IQR). X-Axis reflects the Cluster Number (1-red; 2-black; 3- blue). Y-Axis shows perfusion and oxygenation parameters. (A) ruSO_2_ – intestinal, periumbilical absolute oxygen saturation; (B) ruFlowAU – intestinal, periumbilical blood flow; (C) ruHbAU – intestinal, periumbilical local hemoglobin amount; (D) rlSO2 – liver (V. portae) absolute oxygen saturation; (E) rlFlowAU – liver (V. portae) blood flow; (F) rlHbAU – liver (V.portae) local hemoglobin amount; (G) ResistanceIndexRI – Doppler interrogation of superior mesenteric artery resistance index; (H) PulsatilityIndexPI – Doppler interrogation of superior mesenteric artery pulsatility index. P-values result from Wilcoxon tests and applied oxygenation and perfusion parameters according to the three clusters. P-values here give rather the probability to see a more extreme result of the statistical test function within our sample subset than that actually proving the null hypothesis to be true. Thus, the result gives hardly any clue towards generalization.
